# Analysis of the Adherence and Safety of Second Oral Glucose-Lowering Therapy in Routine Practice From the Mediterranean Area: A Retrospective Cohort Study

**DOI:** 10.3389/fendo.2021.708372

**Published:** 2021-07-14

**Authors:** Bogdan Vlacho, Manel Mata-Cases, Xavier Mundet-Tudurí, Joan-Antoni Vallès-Callol, Jordi Real, Magi Farre, Xavier Cos, Kamlesh Khunti, Dídac Mauricio, Josep Franch-Nadal

**Affiliations:** ^1^ Grup de Recerca Epidemiològica en Diabetis des de l'Atenció Primària (DAP-CAT) Group, Unitat de Suport a la Recerca Barcelona, Fundació Institut Universitari per a la recerca a l’Atenció Primària de Salut Jordi Gol i Gurina (IDIAPJGol), Barcelona, Spain; ^2^ Clinical Trials Unit, Germans Trias i Pujol Health Science Research Institute (IGTP), Barcelona, Spain; ^3^ Pharmacology Department, Universitat Autònoma de Barcelona (UAB), Cerdanyola del Vallès, Spain; ^4^ CIBER of Diabetes and Associated Metabolic Diseases (CIBERDEM), Instituto de Salud Carlos III (ISCIII), Madrid, Spain; ^5^ Departament of Medicine, Universitat Autònoma de Barcelona, Bellaterra, Spain; ^6^ Clinical Pharmacology Unit, Hospital Universitari Germans Trias i Pujol, Badalona, Spain; ^7^ Primary and Hospital Innovation Department, Innovation Office at Institut Català de la Salut, Barcelona, Spain; ^8^ Diabetes Research Centre, College of Life Sciences, University of Leicester, Leicester, United Kingdom; ^9^ Department of Endocrinology and Nutrition, Hospital Universitari de la Santa Creu i Sant Pau, Universitat Autònoma de Barcelona, Bellaterra, Spain; ^10^ Departament of Medicine, University of Vic - Central University of Catalonia, Vic, Spain

**Keywords:** adherence - compliance – persistence, glycemia control, type 2 diabetes, primary care, observational study

## Abstract

The aims of our study was compare adherence measured by the medical possession ratio (MPR), time until discontinuation and describe adverse events after adding a DPP-4i, SGLT-2i, or sulfonylureas (SU) to metformin in a primary care population with insufficient glycemic control. We used routinely-collected health data from the SIDIAP database. The included subjects were matched by propensity score. The follow-up period was up to 24 months or premature discontinuation. The primary outcomes were the percentage of subjects with good adherence, treatment discontinuation and adverse events among treatment groups. The proportion of patients with good adherence (MPR> 0.8) after the addition of DPP-4i, SGLT-2i or SU was 53.6%, 68.7%, and 43.0%, respectively. SGLT-2i users were 1.7 times more likely to achieve good adherence compared with DPP-4i users (odds ratio [OR]:1.72, 98% confidence interval [CI]:1.51, 1.96), and 2.8 times more likely compared with SU users (OR: 0.35, 98% CI: 0.07, 0.29). The discontinuation hazard ratios were 1.43 (98%CI: 1.26; 1.62) and 1.60 (98%CI: 1.42; 1.81) times higher among SGLT-2i and SU users than DPP-4i users during the follow-up period. No differences were observed for adverse events among the treatment groups. In conclusion, in our real-world setting, the combination of SGLT-2i with metformin was associated with better adherence. The mean time until discontinuation was longer in the SGLT-2i group in comparison with the DPP-4i or SU groups.

## Introduction

Good quality management of type 2 diabetes mellitus (T2DM) involves a combination of changes in lifestyle and pharmacological interventions to achieve target glycated hemoglobin (HbA1c) and, thus, reduced risk of macrovascular and microvascular complications (1). However, over time, insulin secretory capacity declines, and most people with T2DM will require escalation of pharmacotherapy to achieve good metabolic control (2). This is common in real clinical practice where first-line treatment with metformin will, in time, require intensification with a second antidiabetic drug to achieve good glycemic control (3–5). According to the current therapeutic guidelines, the selection of a second antidiabetic drug should be based on patient-specific treatment goals, presence of comorbidities, and drug characteristics (6–10). Unfortunately, intensification with additional antidiabetic drugs is often delayed, leaving patients with prolonged periods of poor glycemic control with worse long term outcomes (11). We recently reported a lack of treatment intensification in 1 in 5 patients with HbA1c values >8% (12) and only 20% of the persons with T2DM were treated with dual antidiabetic therapy (13).

There are a number of complex barriers to the proper implementation of antidiabetic treatment both on the healthcare professionals’ and patients’ side. Adherence to pharmacological treatment plays an important role in achieving treatment goals (2). Moreover, data from a meta-analysis suggests that good adherence to antidiabetic treatment was associated with a lower hospitalization rate and all-cause mortality among the persons with T2DM (14). Treatment adherence and persistence are similar, yet distinct, measurements of the degree to which a patient continues treatment after initiation (15). Adherence is defined as “the extent to which a patient acts following the prescribed interval and dosing regimen” (16). Treatment persistence is defined as the length of time from initiation until discontinuation of therapy (16), measured by the drug’s availability, expressed as the continuous filling of prescriptions (17).

Evidence suggests that adherence to medication in T2DM is less than optimal, and many patient factors could influence it, such as comprehension of the treatment regimen and its benefits, emotional well-being, regimen complexity, medication cost and adverse events (18). RWE (real-world evidence) studies have shown that non-adherence to oral antidiabetic drugs is frequent: over 50% in the first year and even higher at the two-year follow-up (19). Low adherence may explain, at least in part, the efficacy gap in the reduction of HbA1c between RWE studies and randomized clinical trials (RCT) (20).

In RWE studies, where prescription or pharmacy claims data are available, adherence is usually measured through the medication possession ratio (MPR), where a value of 0.80 (80%) is the cut-off point that stratifies adherent and non-adherent patients (21). Results from a recently published meta-analysis confirm the high variability in adherence (38.5 to 93.1%) among different observational studies (2). In another meta-analysis, the proportion of adherent patients was found to be suboptimal (67.9%), while the persistence to initial oral antihyperglycemic agents ranged from 41.0% to 81.1% (22). Adverse events can directly influence adherence and persistence to antidiabetic treatment. Hypoglycemias associated with sulphonylureas (SU) and genital tract infections associated with sodium-glucose Cotransporter 2 Inhibitors (SGLT-2i) combined with metformin were the most frequently reported adverse events in recently published meta-analyses (23–25).

We previously published efficacy results regarding the addition of dipeptidyl peptidase-4 inhibitor (DPP-4i), SGLT-2i, or SU as second-line therapies to metformin, showing that users initiating SGLT-2i in combination with metformin achieved greater reduction in weight and combined target HbA1c (≥0.5%) and weight (≥3%) reduction among the cohorts (26). In the present study, we assessed adherence using the MPR and time till discontinuation of DPP-4i, SGLT-2i, or SU added to metformin in subjects with T2DM with insufficient glycemic control in a primary care setting. Additionally, we described the adverse events associated with these drug combinations.

## Material and Methods

### Study Design and Data Source

This was a retrospective cohort study to compare subjects initiating add-on treatment with DPP-4i, SGLT-2i, or SU to metformin. Exposure to these drugs was defined if the user had more than one drug dispensation/prescription register for the first time between January 1st, 2010, and December 31st, 2017. Subjects were followed up for a period of 24 months or until premature discontinuation.

Data were obtained from the primary care SIDIAP database (The Information System for the development of Primary Care Research) (27). This database contains anonymized data from electronic medical records of the people attended in the 279 Primary Care Teams that belong to the Catalan Health Institute, Catalonia, Spain. The Institute’s assigned population is about 5,835,000 individuals (75% of the total Catalan population). Furthermore, the SIDIAP database incorporates laboratory data, prescriptions, and data on drug dispensations extracted from pharmacy-billing records provided by the Catalan Health Service (CatSalut). The SIDIAP database has been extensively used for other epidemiologic and pharmacoepidemiologic national and international research studies, and it is established as a well-validated primary care Spanish database for the study of diabetes (28, 29).

### Inclusion and Exclusion Criteria

Patients were included if they were 18 years or older, diagnosed with T2DM (ICD-10: E11), and had poor glycemic control (HbA1c ≥7%). We defined the inclusion date when the second add-on treatment (DPP-4i, SGLT-2i, or SU) was introduced to metformin for the first time. For each treatment group, we identified drug exposure (index medication) using ATC codes (Anatomical Therapeutic Chemical classification system) from the World Health Organization (WHO) (30), the date of prescription and dispensation. Patients registered with other types of diabetes such as diabetes mellitus type 1, gestational or secondary (ICD-10: E8, E9, E10, O24, E13), and those subjects with missing baseline values for HbA1c and weight were excluded. Subjects could enter the study groups only once.

### Study Variables

At inclusion, we collected routine information on the social-demographic characteristics of subjects (age, gender and toxic habits) and clinical characteristics such as laboratory and clinical parameters related to diabetes control and comorbidities. We collected information about drug prescriptions in each treatment group, dispensations, and both adverse events and discontinuation events during the follow-up period.

#### Outcomes

Adherence was estimated using the medication possession ratio (MPR), calculated as the number of days covered by dispensation divided by the number of days covered by prescription, which is defined as days between the date of initiation of index medication and discontinuation event or up to 24 months. MPR is a validated and standard method to evaluate adherence in studies with routinely-collected health data; good adherence was defined as an MPR value >0.8 (>80%), whereas poor adherence was defined as an MPR value ≤0.8 (≤80%) (21).

Persistence was defined as the time between index treatment initiation and the first discontinuation event. For this study, we considered treatment discontinuation events if there was any gap of at least 90 days (15) without index medication dispensation, any changes in antidiabetic treatment, death, or moving to another healthcare provider. We calculated the proportion of subjects who discontinued treatment for each treatment group at 6, 12, and up to 24 months of follow-up period.

Adverse events were classified into eight categories based on the affected system organ class (SOC) (metabolic, gastrointestinal, hepatic, renal, musculoskeletal, dermatological, hematological, and genitourinary events); these SOCs were chosen as they are the most frequently reported adverse reactions in the summary of product characteristics for each drug group. We described the mortality events (any cause) for the three groups during the follow-up period.

### Statistical Methods

#### Propensity Score Matching

The matching criteria were the same as for the previously published effectiveness analysis related to changes in glycated hemoglobin (HbA1c) and the effect on body weight following the addition of DPP-4i, SGLT-2i, or SU as second-line therapies to metformin (26). The three treatments groups were matched for the following baseline characteristics: weight, HbA1c, sex, age, diabetes duration, year of inclusion, and kidney function. Matching was done by the “Nearest Neighbor algorithm” (caliper=0.01), using the “MatchIt” library of the R (v3.6.1) statistical package (31).

#### Main Analysis

The MPR and persistence were described by mean, standard deviation, median and interquartile range, while good and poor adherence and adverse events were reported by frequency and percentage. We used linear regression models to analyze the differences in MPR as an interval variable among the three treatment groups. The associations between good/poor adherence among the treatment groups were analyzed by logistic regression models, summarized as odds ratios (OR), with 98% confidence intervals (CI). All pairwise comparisons (2X2) was conducted between the three groups, where the family significance level (alpha=0.05) was corrected for multiple paired groups (Bonferroni correction), so the individual test was prefixed at 0.017, and the confidence level at 98%. To analyze the time to a discontinuation event, we used Cox proportional hazards analysis, and hazard ratio (HR), CI, and p-value were summarized. We used Kaplan-Meier curves to graphicly visualize treatment persistence up to 24 months of the observation period in each treatment group. As a sensitivity analysis, adjusted estimates were calculated with multivariable models. The variables used for adjustment were age, sex, number of comorbidities, weight, HbA1c, year of inclusion, duration of diabetes, and glomerular filtration rate. The statistical analyses were performed using R3.6.1 software (https://www.r-project.org/).

### Ethical Review

The study was approved by the Ethics Committee of the Primary Health Care University Research Institute (IDIAP) Jordi Gol, Barcelona (approval code: P17/205).

## Results

### Patient Characteristics

A total of 75,808 poorly controlled T2DM subjects initiating a second antidiabetic drug in addition to metformin were included: 27,878 (36.7%) initiated a DPP-4i, 2,198 (2.89%) a SGLT-2i and 45,732 (60.3%) an SU. The study flow chart is shown in **Supplementary Figure 1**. After matching, 6,310 subjects were compared: 2,124 for DPP-4i, 2,124 for SGLT-2i and 2,062 for SU (**Supplementary Figure 2**). The baseline characteristics of subjects in each study group are shown in **Supplementary Table 1**. Overall, the mean age was 60.8 years ( ± 11.7), with a mean diabetes duration of 7.61 years ( ± 6.59), and an HbA1c of 8.8% ( ± 1.45) (72.3 mmol/mol ( ± 15.9). Subjects in the DPP-4i group were older with a mean age of 61.2 ( ± 12.1), while those in the SGLT-2i treatment group had a longer diabetes duration of 7.89 ( ± 6.67) and had a higher BMI 33.9 ( ± 5.80) compared to the other groups. SGLT-2i users also had slightly higher triglycerides and a worse comorbidity profile, especially for cardiovascular complications. The baseline characteristics and analysis of effectiveness among the three treatment groups have been recently published (26).

### Adherence to Treatment

**Table 1** summarises the data related to adherence and drug dispensations. Comparison between study groups showed that good adherence (MPR>0.8) was achieved for most of the SGLT-2i and DPP-4i treated subjects (68.7% and 53.6%, respectively), while the majority of SU users had poor adherence (43.0%).

**Table 1:** Medical possession ratio, adherence, persistence (time until discontinuation and discontinuations) among the three treatment groups.

	MET+ DPP-4i (n = 2113)	MET+SGLT-2i (n = 2117)	MET+ SU (n = 2056)
**Medical possession ratio (MPR)**			
**Medication possession ratio, Mean (SD)**	0.71 (0.34)**	0.78 (0.34)**	0.63 (0.35)**
**Medication possession ratio, Median [IQR: 25th;75th]**	0.86 [0.43;1.00]**	1.00 [0.62;1.00]**	0.64[0.33;1.00]**
**Number medicine packages dispensed, Mean (SD)**	10.6 (8.62)*	10.7 (8.52)*	9.88 (12.9)*
**Poor adherence (≤ 0.8)**	981 (46.4%)**	662 (31.3%)**	1172 (57.0%)**
**Good adherence (>0.8)**	1132 (53.6%)**	1455 (68.7%)**	884 (43.0%)**
**Persistence**			
**Persistence time on treatment, Mean (SD)**	372 (330)**	385 (289)**	343 (306)**
**Persistence time on treatment, Median, [IQR: 25th;75th]**	274 [121;548]**	333 [150;600]**	272 [91.2;486]**
**Discontinuation events**			
**Discontinuation of treatment 6 m: % (98% CI Linf, Lsup)**	12.9 (11.4, 14.5)	18.6 (16.8, 20.4)	21.3 (19.4, 23.2)
**Discontinuation of treatment 12 m: % (98% CI Linf, Lsup)**	20.1(18.0, 22.0)	28.6 (26.4, 30.7)	32.1 (29.7, 34.3)
**Discontinuation of treatment 24 m: % (98% CI Linf, Lsup)**	28.8 (26.0, 31.4)	39.7(36.8, 42.5)	43.0 (39.8, 48.9)

CI, confidence interval; DPP-4i, dipeptidyl peptidase-4 inhibitors; IQR, inter-quartile range; Linf, inferior limit; Lsup, superior limit; m, months; MET, metformin; SD, standard deviation; SGLT-2i, sodium/glucose cotransporter 2 inhibitors; SU, sulphonylureas; *p-value =0.018; **p-value <0.001.

**Supplementary Table 2** shows adherence for different drug within the drug groups. Alogliptin in combination with metformin had the highest mean MPR in the DPP-4i group (0.81 ± 0.28), canagliflozin in combination with metformin in the SGLT-2i group (0.82 ± 0.30), and glimepiride in combination with metformin in the SU group (0.91 ± 0.21). Multiple logistic regression analysis showed that SGLT-2i users were 1.7 and 2.8 times more likely to be associated with good adherence than DPP-4i users (adjusted OR: 1.72, 98% CI: 1.51, 1.96), or SU users (adjusted OR: 0.35, 98% CI: 0.07, 0.29), respectively.

The DPP-4i users were 1.6 times more likely to be associated with good adherence than SU users (OR: 0.59, 98% CI: 0.52, 0.67). A mean difference in MPR of 6% was observed between SGLT-2i users and DPP-4i users (adjusted DR: 0.06, 98% CI: 0.04, 0.08) and 14% compared with SU users (adjusted DR: -0.14, 98% CI: -0.16, -0.11); the difference was 8% between DPP-4i and SU users (adjusted DR: -0.08, 98% CI: -0.10, -0.06). Comparing the difference in number of packages dispensed between groups, we only observed statistical differences between SU and DPP-4i users (1.02 fewer packages in the former group; adjusted DR: -1.02, 98% CI: -1.59, -0.46). The odds ratios for good adherence, MPR differences and the number of dispensed packages among the treatment groups are shown in **Table 2**.

**Table 2:** Odds ratios for good adherence, MPR differences and number of dispensed packages among the cohorts.

	Good adherence (MPR>0.8)		MPR differences		Number of dispensed packages differences	
	Unadjusted OR(98% CI)	Adjusted OR(98% CI)	Unadjusted DR(98% CI)	Adjusted DR(98% CI)	Unadjusted DR(98% CI)	Adjusted DR(98% CI)
**SGLT-2i+MET, ref: DPP-4i+MET**	1.90 (1.67, 2.16)*	1.72 (1.51, 1.96)*	0.08 (0.06, 0.1)*	0.06 (0.04, 0.08)*	0.13 (-0.49, 0.74)	-0.63 (-1.19,-0.06)
**SU+ MET, ref: DPP-4i+MET**	0.65 (0.57,0.73)*	0.59 (0.52, 0.67)*	-0.07 (-0.09,-0.05)*	-0.08 (-0.10, -0.06)*	-0.71 (-1.33,-0.08)	-1.02 (-1.59,-0.46)*
**SU +MET, ref: SGLT-2i+MET**	0.34 (0.29, 0.40)*	0.35 (0.07, 0.29)*	-0.15(-0.17, -0.13)*	-0.14 (-0.16, -0.11)*	-0.83 (-1.6,-0.08)*	-0.34 (-1.09,0.29)

*Statistically significant p-value (p-value <0.017).

CI, confidence interval; IDPP-4i, dipeptidyl peptidase-4 inhibitors; OR, odds ratio; DR: differences; SGLT-2i, sodium/glucose cotransporter 2 inhibitors; SU, sulphonylureas; MET, metformin.

### Treatment Persistence

**Table 1** summarizes the results of discontinuation and persistence in the 3 study groups. The mean time until discontinuation was longer in the SGLT-2i group in comparison with the DPP-4i or SU groups: 385 ( ± 289), 372 ( ± 330) and 343 ( ± 306) days, respectively. During the initial six month period, 21.3% of SU users discontinued treatment, compared with 18.6% of SGLT-2i users and only 12.9% of DPP-4i users. At the end of the 24-month follow-up period, 43.0% of SU users, 39.7% of SGLT-2i users, and 28.8% of DPP-4i users had ceased treatment.

**Figure 1:**
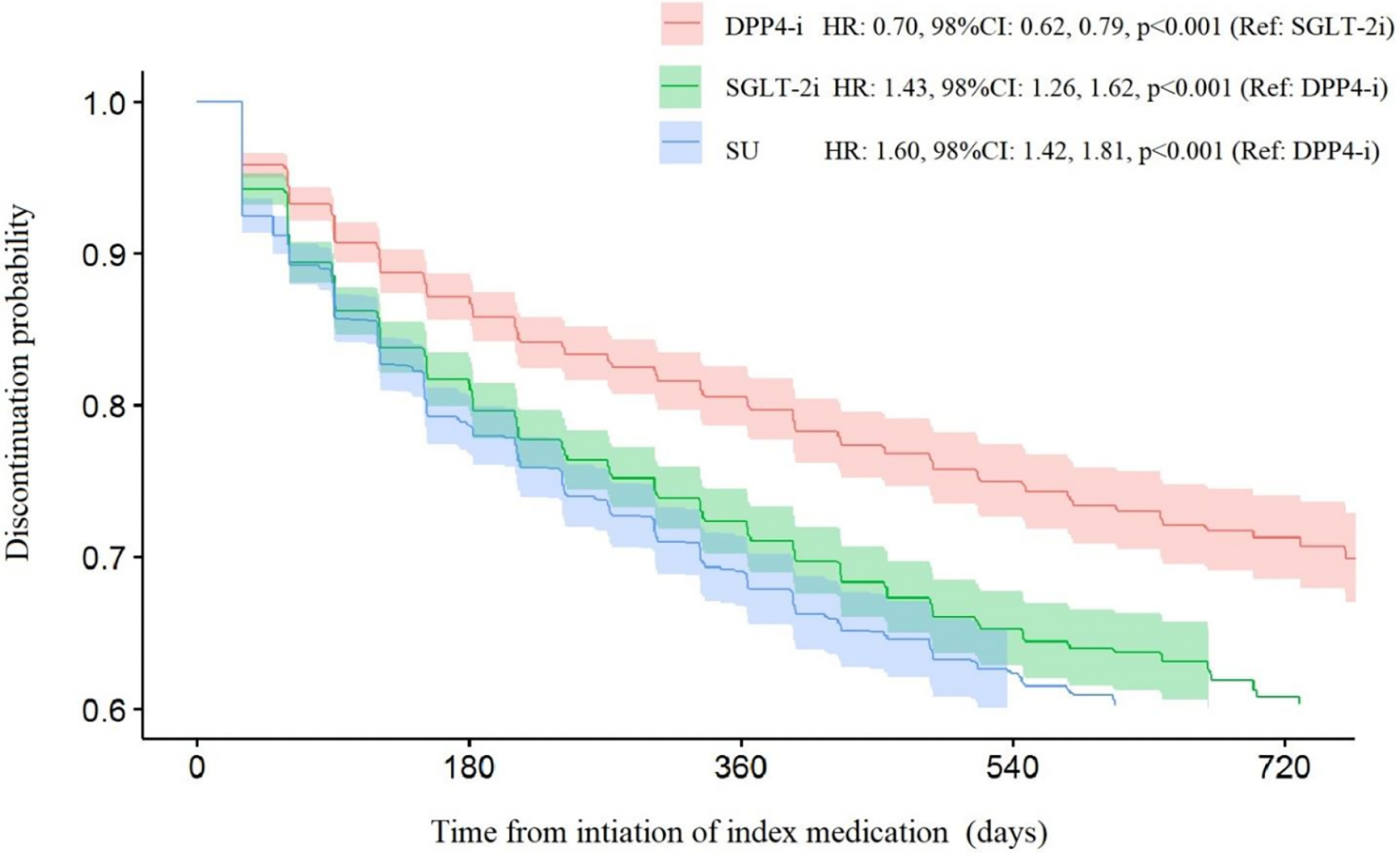
Kaplan-Meier discontinuation probability curves for the three treatment groups.

The Kaplan-Meier curves of persistence are shown in **Figure 1** and summarized in **Supplementary Table 3**. We performed a Cox proportional hazards analysis to compare the hazard risk ratios for discontinuation events. The risk of discontinuation was 1.4 times higher for SGLT-2i (HR: 1.43, 98% CI: 1.26, 1.62) and 1.6 times higher for SU (HR: 1.60, 98% CI: 1.42, 1.81) compared to DPP-4i. Furthermore, the risk of discontinuation among SU users was 1.1 times higher than that of SGLT-2i users (HR: 1.12, 98% CI: 1.00, 1.26).

### Adverse Events

The results for adverse events are reported in **Table 3**. We observed that gastrointestinal, musculoskeletal, dermatological, and urogenital were the most frequent adverse events during the follow-up period in all three groups. In the SGLT-2i group, urogenital, metabolic and dermatological adverse reactions were more frequent than in the other treatment groups (10.5%, 0.19% and 3.01%, respectively) but without statistically significant differences between groups. There were no significant differences in the frequency of gastrointestinal adverse events between the groups.

**Table 3:** Adverse events among the treatment groups.

Adverse event, n (%)	MET+ DPP-4i (n = 2113)	MET+SGLT-2i (n = 2117)	MET+ SU (n = 2056)
**Metabolic adverse event**	0 (0.00%)	4 (0.19%)	0 (0.00%)
**Gastrointestinal adverse events**	156 (7.34%)	149 (7.02%)	145 (7.03%)
**Hepatic adverse events**	26 (1.22%)	20 (0.94%)	23 (1.12%)
**Kidney adverse events**	25 (1.18%)	28 (1.32%)	12 (0.58%)
**Musculoskeletal system adverse events**	57 (2.68%)	57 (2.68%)	48 (2.37%)
**Dermatological adverse events**	54 (2.54%)	64 (3.01%)	49 (2.38%)
**Hematological adverse events**	1 (0.05%)	2 (0.09%)	0 (0.00%)
**Urogenital adverse events**	161 (7.58%)	223 (10.5%)	160 (7.76%)
**Death by any cause**	33 (1.55%)	29 (1.37%)	39 (1.89%)

*No statistically significant difference were observed among the groups; DPP-4i, dipeptidyl peptidase-4 inhibitors; SGLT-2i, sodium/glucose cotransporter 2 inhibitors; SU, sulphonylureas; MET, metformin.

## Discussion

In the current study, among 6,310 propensity score-matched users who initiated a second line add-on therapy to metformin with DPP-4i, SGLT-2i or SU in Catalonia, the highest adherence and persistence was observed in SGLT-2i users.

Comparing the adherence among the study groups, 68.7% of users in the SGLT-2i treatment group had good adherence (MPR>0.8), while this percentage was lower in both DPP-4i and SU users (53.6% and 43%, respectively). In an observational study with 11,961 subjects in the US, the percentage of good adherence (MPR≥0.8) for subjects initiating an SGLT-2i was 56.2-58.8% for canagliflozin, 36.4-36.7% for dapagliflozin and 45.7% for sitagliptin after 12 months (15). Our study showed a similar tendency, although with a higher level of good adherence: canagliflozin 62.5-73.7%, dapagliflozin 71.2-71.7%, and sitagliptin 49.9-51.8%. In an observational study with 171,220 T2DM subjects from Sweden during 2005 and 2006, the refill adherence for dual therapy with SUs (glibenclamide, glipizide, glimepiride) was high (91.3%, 91.0%, and 91.7%, respectively) (32), however the proportion of subjects with good adherence in our study was lower for the same drugs (50%, 85.3% and 44.4%, respectively).

Our results show that SGLT-2i users were more likely to have good adherence to treatment than DPP-4i and SU users (1.7 and 2.8 times higher, respectively). Results from an administrative-claims study in the US reported that patients who initiated an SGLT-2 inhibitor were 1.36 times more likely to be adherent to their medication and 1.35 times less likely to discontinue their medication than patients who initiated an SU (33). In another RWE study from the US, comparing the DPP-4i sitagliptin with SUs as an add-on therapy to metformin, subjects in the SU group had lower adherence and persistence (34). Similar findings were observed in a retrospective RWE study with 238,372 subjects, where DPP-4i users had a significantly greater OR of being adherent than users initiating an SU; the authors pointed to a better tolerability profile of DPP-4i as an explanation of their findings (19).

About 79.9% of DPP-4i users, 71.4% of SGLT-2i users, and 67.9% of SU users persisted with their initial therapy during the first year of treatment in our study. In an RWE study by Farr et al. (19), the authors reported that over 40% of SU initiators stopped refiling in the first year. In another RWE study from Hungary, the persistence rate after the first 12 months was 69.6% for DPP-4i users and 67.8% for SGLT-2i users (35). A meta-analysis of previous studies of treatment persistence to oral antidiabetic drugs reported persistence rates ranging from 33 to 61%, with an overall mean percentage of persistence of 49.2% (95% CI: 40.1%– 58.3%) among the studies that investigated only persistence to the index medication (22). We found that the risk of treatment discontinuation among SGLT-2i and SU users compared with DPP-4i users during the follow-up period was 43% and 60% higher, respectively. These results are in line with other RWE studies, where the risk of discontinuation was 40% higher among SU initiators (adjusted HR: 1.390, 95% CI: 1.363, 1.418) (19) and 6% higher among SGLT-2i users (HR: 1.066, 95% CI: 1.036–1.096) (35) compared with DPP-4i users. High discontinuation rates and poor adherence are important factors that may induce possible issues with the initially prescribed treatment. In the current study, among the users who initiated SU in combination with metformin, despite the relatively large gap period (90 days) without dispensation, two of ten subjects stopped the initial treatment during the initial six months. On the other hand, in the SGLT-2i group, the percentage of users with good adherence was higher, but discontinuation rates were higher than in the DPP-4i users. A possible explanation for this could be an improved tolerability in the DPP-4i group (36).

With regards to safety, the most frequently reported adverse events were gastrointestinal and urogenital disturbances. We found higher percentages of urogenital, metabolic and dermatological adverse events in the SGLT-2i group and more frequent gastrointestinal events in the DPP-4i group but without significant differences. Indeed, it is well reported that SGLT-2i drugs are often associated with a higher incidence of urogenital infections (mycotic genital infections such as vaginitis in women and balanitis in man) (37–39). However, despite the occurrence of these adverse effects, these episodes are often regarded as mild by patients (40); additionally, their incidence tends to decline over time without the need for halting SGLT-2i therapy (41).

Studies have previously shown that achieving better adherence is associated with improved glycemic control (17), but many factors could influence adherence and persistence to treatment. The patient is the primary driver of treatment adherence and may be influenced by both efficacy and tolerability; a subject who experiences undesired side effects of medication is less likely to take the prescribed drug (15). One UK study reported that gastrointestinal side effects, hypoglycemia, weight change, and efficacy were the most important factors determining patient preferences for oral antidiabetic drugs (42).

In our study, efficacy in combination with weight reduction could be the reason for good adherence and persistence among the SGLT-2i and DPP-4i users. Our previous study showed that the addition of SGLT-2i or DPP-4i to metformin was associated with a greater weight reduction (3.47 kg and 1.21 kg, respectively) (26). Additionally, the proportion of subjects who achieved a combined target of HbA1c (≥0.5%) and weight (≥3%) reduction was greater in these two treatment groups (26). Previous studies have shown that the presence of certain conditions, such as depression and mental disorders, before the first antidiabetic drug prescription is associated with non-persistence to antidiabetic treatment (43). In our study, the lowest persistence and adherence were observed among SU users; however, at baseline, these users had fewer mental disorder comorbidities than the users included in the other treatment groups.

There are some limitations to our study. As per the study design, we only included subjects with complete data for baseline HbA1c and weight; the study population is a highly selected sample which potentially diminishes the external validity. Moreover, we cannot rule out that patients having both variables at baseline were treated more proactively to favor a better T2DM control; however, due to the matching process, we would expect this limitation to be the same for the three study groups. Another limitation is the relatively small sample size to observe the number of adverse events, mainly due to the propensity score matching, which drastically reduces the population size and, thus, the total number of adverse events. However, our goal was to describe the number of events among the treatment groups as opposed to analyzing statistically significant differences. Finally, the retrospective nature of the study precludes explaining the reason for treatment discontinuation, so we are not able to discern whether the differences in adherence could be due to the drug itself, to the risk of adverse events, to the number of pills per day or to the use of available fixed-dose combinations. Strengths of our study include a population-based cohort, long follow-up of two years, propensity matching and outcomes for adherence, persistence and adverse events.

In conclusion, the results of the present study show better drug adherence and longer persistence among subjects on SGLT-2i as an add-on to metformin compared with DPP-4i or SU users. Subjects being treated with DPP-4i combined with metformin had the fewest discontinuation events during the follow-up period. These results may help clinicians better understand the treatment trajectory following the addition of DPP-4i, SGLT-2i, or SU to metformin. However, further studies in real-world conditions are needed to identify factors related to good adherence, persistence and safety amongst these three commonly prescribed drug combinations.

## Data Availability Statement

The data analyzed in this study is subject to the following licenses/restrictions: The data controller for SIDIAP does not allow the sharing of raw data. The source code is available at https://github.com/jrealgatius/METPLUS. Requests to access these datasets should be directed to JF-N, dap.cat.info@gmail.com.

## Ethics Statement

The studies involving human participants were reviewed and approved by Ethics Committee of the Primary Health Care University Research Institute (IDIAP) Jordi Gol, Barcelona (approval code: P17/205). Written informed consent for participation was not required for this study in accordance with the national legislation and the institutional requirements.

## Author Contributions

JF-N, MM-C, JR, DM, XM-T, JAV-C, and BV conceived the research and participated in its design. JR performed the statistical analysis. BV wrote the initial draft of the manuscript, which JF-N, MM-C, JR, DM, XM-T, JAV-C, XC MF and KK edited. All authors contributed to the article and approved the submitted version.

## Funding

This study received funding from AstraZeneca, Spain [grant number ESR-16-12628]. The funder was not involved in the study design, collection, analysis, interpretation of data, the writing of this article or the decision to submit it for publication.

## Conflict of Interest

MM-C has received an advisory and or speaking fees from Astra-Zeneca, Bayer, Boehringer Ingelheim, GSK, Lilly, MSD, Novartis, Novo Nordisk, and Sanofi; he has received research grants to the institution from Astra-Zeneca, GSK, Lilly, MSD, Novartis, Novo Nordisk, and Sanofi. JF-N has received advisory and or speaking fees from Astra-Zeneca, Ascensia, Boehringer Ingelheim, GSK, Lilly, MSD, Novartis, Novo Nordisk, and Sanofi; he has received research grants to the institution from Astra-Zeneca, GSK, Lilly, MSD, Novartis, Novo Nordisk, Sanofi, and Boehringer. DM has received advisory and/or speaking fees from Astra-Zeneca, Ascensia, Boehringer Ingelheim, GSK, Lilly, MSD, Novartis, Novo Nordisk, and Sanofi; he has received research grants to the institution from Astra-Zeneca, GSK, Lilly, MSD, Novartis, Novo Nordisk, Sanofi, and Boehringer. KK has acted as a consultant, speaker or received grants for investigator-initiated studies for Astra Zeneca, Novartis, Novo Nordisk, Sanofi-Aventis, Lilly and Merck Sharp & Dohme, Boehringer Ingelheim, Bayer, Berlin-Chemie AG/Menarini Group, Janssen, and Napp.

The remaining authors declare that the research was conducted in the absence of any commercial or financial relationships that could be construed as a potential conflict of interest.
