# Supplementary material for: Analysis of the Adherence and Safety of Second Oral Glucose-Lowering Therapy in Routine Practice From the Mediterranean Area: A Retrospective Cohort Study
Source: Front Endocrinol (Lausanne). 2021 Jul 14;12:708372. doi: 10.3389/fendo.2021.708372 (PMC8318034; doi:10.3389/fendo.2021.708372)
Supplement: Supplementary file 2 [file DataSheet_2.pdf]

ONLINE-ONLY SUPPLEMENTARY MATERIALS

The authors have provided these supplemental materials to give the readers additional information about the study.

Analysis of the adherence and safety of second oral glucose-lowering therapy in routine practice from the Mediterranean area: A retrospective cohort study

Bogdan Vlacho, Manel Mata-Cases, Xavier Mundet-Tudurí, Joan Antoni Vallès-Callol, Jordi Real, Magí Farré, Xavier Cos, Kamlesh Khunti, Dídac Mauricio, Josep Franch-Nadal

TABLE OF CONTENTS

		PAGE
**Supplementary Figure 1**	Flow chart of the patients included in the study	2
**Supplementary Figure 2**	Balancing for baseline variables before and after propensity-score matching across the different treatment cohorts	3
**Supplementary Table 1**	Baseline characteristics of the study population for users initiating second add-on treatment to metformin with DPP-4i, an SGLT-2i, or an SU	4-5
**Supplementary Table 2**	Medical possession ration for each cohort drug	6
**Supplementary Table 4**	Hazard ratios for discontinuation events among the cohorts	7

Supplementary Figure 1. Flow chart of the patients included in the study

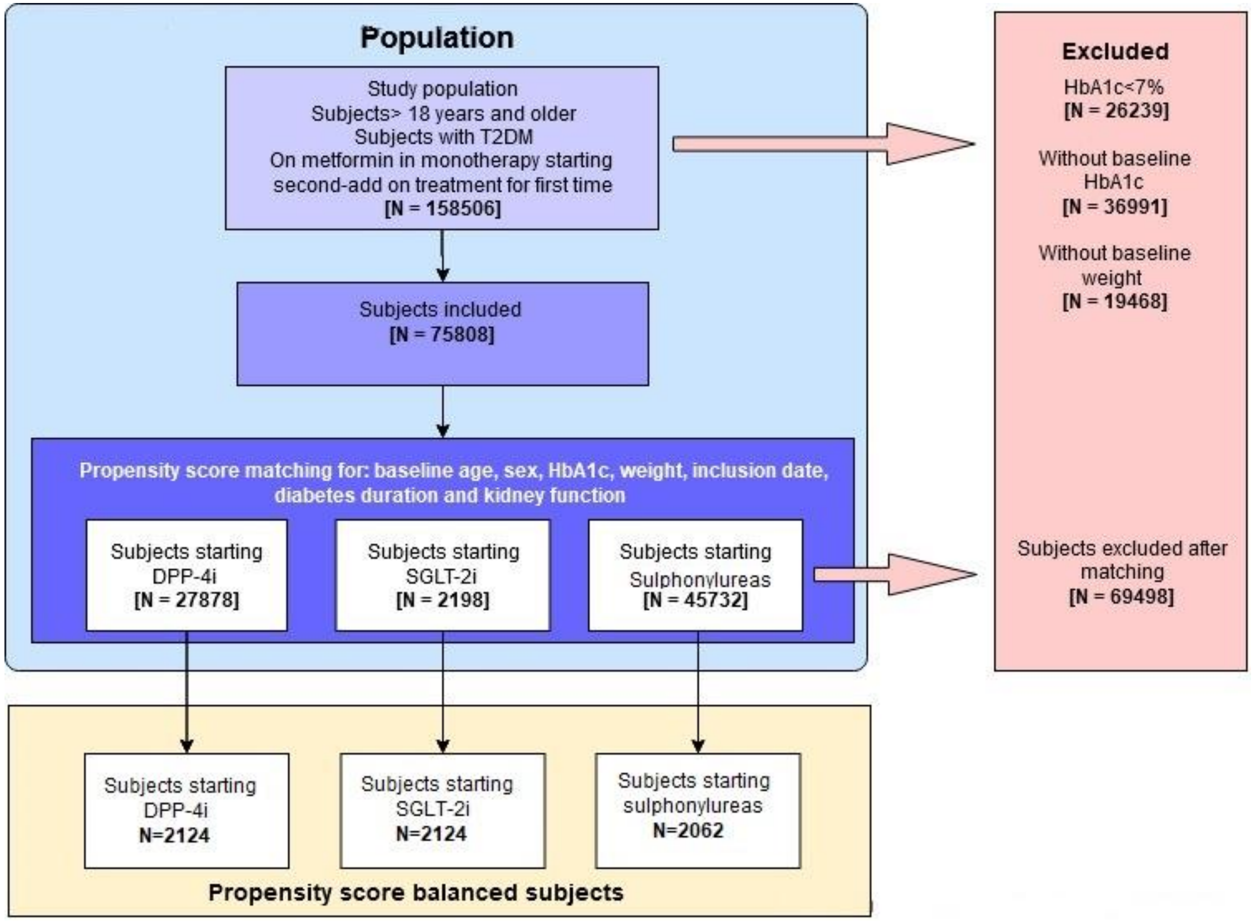

HbA1c, glycated hemoglobin; DPP-4i, dipeptidyl peptidase-4 inhibitors; MET, metformin; SGLT-2i, sodium/glucose cotransporter 2 inhibitors; SU, sulphonylureas

Supplementary Figure 2. Balancing for baseline variables before and after propensity-score matching across the different treatment cohorts

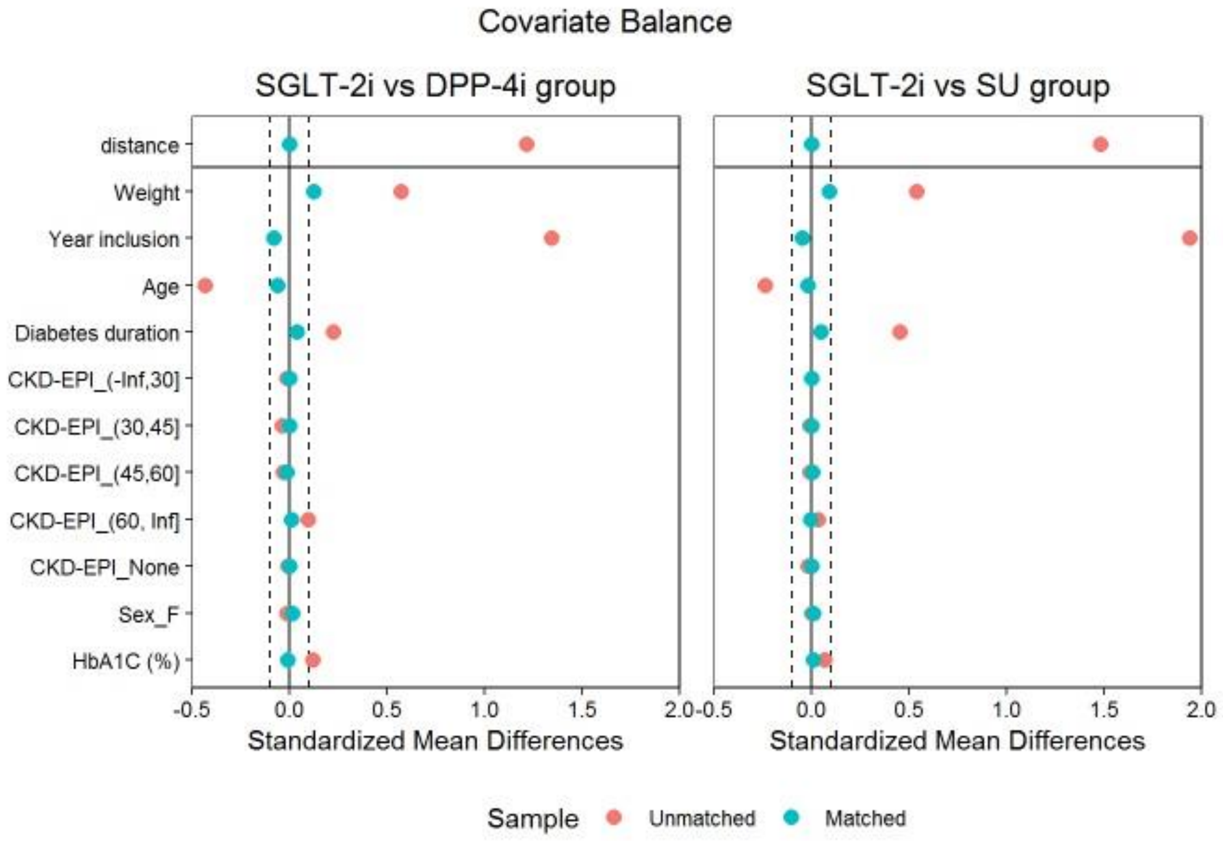

HbA1c, glycated hemoglobin; CDK-EPI, estimated glomerular filtration rate (CDK-EPI formula); F, female; DPP-4i, dipeptidyl peptidase-4 inhibitors; MET, metformin; SGLT-2i, sodium/glucose cotransporter 2 inhibitors; SU, sulphonylureas

Supplementary Table 1. Baseline characteristics of the study population for users initiating second add-on treatment to metformin with DPP-4i, an SGLT-2i, or an SU.

Characteristic	Met+DPP-4i (n = 2124)	Met+SGLT-2i (n = 2124)	Met+ SU (n = 2062)
**Age, mean (SD), years**	61.2 (12.1)	60.5 (11.1)	60.6 (12.0)
**Gender, n (%)**			
**Female**	892 (42.0)	924 (43.5)	868 (42.1)
**Male**	1232 (58.0)	1200 (56.5)	1194 (57.9)
**Smoking habit, n (%)**			
**Non-smoker**	1010 (47.9)	957 (45.3)	996 (48.5)
**Ex-smoker**	700 (33.2)	758 (35.9)	616 (30.0)
**Smoker**	398 (18.9)	398 (18.8)	440 (21.4)
**Clinical variables**			
**Diabetes duration, mean (SD), years**	7.64 (7.12)	7.89 (6.67)	7.28 (5.89)
**HbA1c, mean (SD), %**	8.78 (1.48)	8.77 (1.49)	8.75 (1.39)
**HbA1c, mean (SD), mmol/mol**	72.3 (15.9)	72.5 (16.2)	72.4 (16.2)
**Weight, mean (SD), kg**	89.2 (18.5)	91.4 (17.5)	89.5 (18.2)
**BMI, mean (SD), kg/m^2**	32.8 (5.72)	33.9 (5.80)	32.8 (5.87)
**Blood pressure, mean (SD), mmHg**			
**SBP**	134 (14.4)	133 (13.8)	134 (13.5)
**DBP**	78.8 (10.2)	78.0 (9.55)	79.4 (9.46)
**Renal function**			
**Albumin / creatinine ratio, mean (SD)**	57.1 (217)	72.0 (254)	43.4 (145)
**eGFR, mean (SD), mL/min/1.73 m^2**	81.2 (13.4)	81.0 (13.0)	82.2 (12.9)
**eGFR groups (mL/min/1.73 m^2), n (%)**			
**< 30**	5 (0.24)	6 (0.28)	10 (0.48)
**30 – 44**	31 (1.46)	36 (1.69)	34 (1.65)
**45 – 59**	182 (8.57)	155 (7.30)	140 (6.79)
**≥ 60**	1871 (88.1)	1892 (89.1)	1845 (89.5)

Characteristic	Met+DPP-4i (n = 2124)	Met+SGLT-2i (n = 2124)	Met+ SU (n = 2062)
**Lipid profile, mean (SD), mg/dL**			
**Total Cholesterol**	191 (45.2)	186 (44.2)	193 (41.4)
**HDL-cholesterol**	46.7 (11.3)	46.2 (12.1)	46.9 (11.5)
**LDL-cholesterol**	106 (33.3)	102 (34.1)	108 (33.0)
**Triglycerides**	207 (152)	216 (180)	207 (167)
**Number of comorbidities, mean (SD)**	1.86 (1.22)	2.05 (1.25)	1.76 (1.16)
**Comorbidities, n (%)**			
**Heart failure**	89 (4.19)	104 (4.90)	73 (3.54)
**Peripheral vascular disease**	79 (3.72)	116 (5.46)	77 (3.73)
**Stroke**	125 (5.89)	156 (7.34)	99 (4.80)
**Ischemic heart disease**	205 (9.65)	334 (15.7)	164 (7.95)
**Hypertension**	1426 (67.1)	1527 (71.9)	1363 (66.1)
**Hyperlipidemia**	1220 (57.4)	1318 (62.1)	1193 (57.9)
**Diabetic neuropathy**	77 (3.63)	155 (7.30)	42 (2.04)
**Diabetic retinopathy**	179 (8.43)	350 (16.5)	116 (5.63)
**Acute/chronic pancreatitis**	29 (1.37)	53 (2.50)	19 (0.92)
**COPD**	160 (7.53)	167 (7.86)	136 (6.60)
**Renal failure**	159 (7.49)	140 (6.59)	124 (6.01)
**Relevant liver disease (excluding steatosis)**	75 (3.53)	86 (4.05)	47 (2.28)
**Major Mental disorders**	431 (20.3)	422 (19.9)	375 (18.2)

BMI, body mass index; DBP, diastolic blood pressure; eGFR, estimated glomerular filtration rate (CDK-EPI formula); HbA1c, glycated hemoglobin A1c; SBP, systolic blood pressure; SD, standard deviation; HDL, high-density lipoprotein; LDL, low-density lipoprotein; COPD, chronic obstructive pulmonary disease; DPP-4i, dipeptidyl peptidase-4 inhibitors; SGLT-2i, sodium/glucose cotransporter 2 inhibitors; SU, sulphonylureas; MET, metformin

Supplementary Table 2. Medical possession ratio for each cohort drug

	Drugs	Number of subjects	MPR (mean)	MPR (SD)	Number of subjects with MPR >0.8	Percentage of subjects with MPR >0.8
MET+DPP-4i	Metformin and sitagliptin fixed-dose	956	0.6905992	0.3366032	477	49.90
	Sitagliptin and metformin	559	0.6928648	0.3479330	290	51.88
	Metformin and vildagliptin fixed-dose	292	0.7563656	0.3205071	178	60.96
	Linagliptin and metformin	99	0.7234033	0.3603052	60	60.61
	Metformin and linagliptin fixed-dose	87	0.8112913	0.2919534	58	66.67
	Vildagliptin and metformin	70	0.7150758	0.3289317	38	54.29
	Metformin and alogliptin fixed-dose	34	0.5928021	0.3713236	13	38.24
	Alogliptin and metformin	22	0.8181361	0.2844784	16	72.73
	Saxagliptin and metformin	3	0.6666667	0.5773503	2	66.67
	Metformin and saxagliptin fixed-dose	2	0.2688730	0.3802439	0	0.00
MET+SGLT-2i	Dapagliflozin and metformin	539	0.8022109	0.3331420	384	71.24
	Metformin and dapagliflozin fixed-dose	521	0.8149182	0.3139571	374	71.79
	Empagliflozin and metformin	501	0.7641476	0.3572087	334	66.67
	Metformin and empagliflozin fixed-dose	284	0.7172564	0.3751410	169	59.51
	Canagliflozin and metformin	175	0.8242860	0.3077732	129	73.71
	Metformin and canagliflozin fixed-dose	104	0.7552019	0.3457346	65	62.50
MET+ SU	Gliclazide and metformin	1716	0.5983946	0.3463613	648	37.76
	Glimepiride and metformin	198	0.9146698	0.2179803	169	85.35
	Glibenclamide and metformin	125	0.6599701	0.3414771	55	44.35
	Glipizide and metformin	23	0.7276963	0.3251168	11	50.00

MPR: Medical possession ratio; DPP-4i, dipeptidyl peptidase-4 inhibitors; SD, standard deviation; SGLT-2i, sodium/glucose cotransporter 2 inhibitors; SU, sulphonylureas; MET, metformin

Supplementary Table 3. Hazard ratios for discontinuation events among the cohorts

	With stopping event N=4305	Without stopping event N=1674	HR (98% CI)	p-value
MET+DPP-4i	1602 (37.2%)	429 (25.6%)	Ref.	Ref.
MET+SGLT-2i	1341 (31.1%)	609 (36.4%)	1.43 (1.26;1.62)	<0.001
MET+ SU	1362 (31.6%)	636 (38.0%)	1.60 (1.42;1.81)	<0.001
MET+DPP-4i	1602 (37.2%)	429 (25.6%)	0.70 (0.62;0.79)	<0.001
MET+SGLT-2i	1341 (31.1%)	609 (36.4%)	Ref.	Ref.
MET+ SU	1362 (31.6%)	636 (38.0%)	1.12 (1.00;1.26)	0.041

CI, confidence interval; IDPP-4i, dipeptidyl peptidase-4 inhibitors; HR, hazard ratio; SGLT-2i, sodium/glucose cotransporter 2 inhibitors; SU, sulphonylureas; MET, metformin
