# Supplementary material for: Analysis of the Adherence and Safety of Second Oral Glucose-Lowering Therapy in Routine Practice From the Mediterranean Area: A Retrospective Cohort Study
Source: Front Endocrinol (Lausanne). 2021 Jul 14;12:708372. doi: 10.3389/fendo.2021.708372 (PMC8318034; doi:10.3389/fendo.2021.708372)
Supplement: Supplementary file 3 [file Table_1.docx]

**Analysis of the adherence and safety of second oral glucose-lowering therapy in routine practice from the Mediterranean area: A retrospective cohort study**

ATC/DDD and ICD code list

| **Type of data** | **Code** | **Description** |
| --- | --- | --- |
| cmbdh_diagnostics | 443 | Other peripheral angiopathies |
| cmbdh_diagnostics | 4438 | Other specified peripheral angiopathies |
| cmbdh_diagnostics | 44389 | Other specified peripheral angiopathies |
| cmbdh_diagnostics | 41406 | Native coronary artery atherosclerosis of transplanted heart |
| cmbdh_diagnostics | 41411 | Coronary vessel aneurysm |
| cmbdh_diagnostics | 4111 | Intermediate coronary syndrome |
| cmbdh_diagnostics | 4143 | Coronary atherosclerosis caused by plaques rich in lipids |
| cmbdh_diagnostics | 41401 | Native coronary artery atherosclerosis |
| cmbdh_diagnostics | 41405 | Coronary atherosclerosis of bypass graft of unspecified type |
| cmbdh_diagnostics | 414 | Other forms of chronic ischemic heart disease |
| cmbdh_diagnostics | 411 | Other acute and subacute forms of ischemic heart disease |
| cmbdh_diagnostics | 41412 | Coronary artery dissection |
| cmbdh_diagnostics | 4139 | Other types of angina and angina not specified |
| cmbdh_diagnostics | 4131 | Prinzmetal's angina |
| cmbdh_diagnostics | 4148 | Other specified forms of chronic ischemic heart disease |
| cmbdh_diagnostics | 4140 | Coronary atherosclerosis |
| cmbdh_diagnostics | 4144 | Coronary atherosclerosis caused by a calcified coronary lesion |
| cmbdh_diagnostics | 41400 | Coronary atherosclerosis of vessel of unspecified type, native or graft |
| cmbdh_diagnostics | 41407 | Coronary atherosclerosis of a graft (arterial) bypass graft (transplanted) |
| cmbdh_diagnostics | 4142 | Chronic total occlusion of the coronary artery |
| cmbdh_diagnostics | 41410 | Cardiac aneurysm (wall) |
| cmbdh_diagnostics | 41402 | Autologous venous graft coronary atherosclerosis |
| cmbdh_diagnostics | 4149 | Unspecified chronic ischemic heart disease |
| cmbdh_diagnostics | 41403 | Non-autologous biological graft coronary atherosclerosis |
| cmbdh_diagnostics | 4130 | Angina decubitus |
| cmbdh_diagnostics | 4141 | Cardiac aneurysm and dissection |
| cmbdh_diagnostics | 413 | Angina pectoris |
| cmbdh_diagnostics | 41404 | Coronary artery graft atherosclerosis |
| cmbdh_diagnostics | 41419 | Other cardiac aneurysms |
| cmbdh_diagnostics | 412 | Old myocardial infarction |
| cmbdh_diagnostics | 24930 | Diabetes mellitus secondary to other types of coma, not mentioned as uncontrolled or not specified |
| cmbdh_diagnostics | 24941 | Diabetes mellitus secondary with renal manifestations, uncontrolled |
| cmbdh_diagnostics | 25041 | Diabetes with renal manifestations, type I [juvenile type], not mentioned as uncontrolled |
| cmbdh_diagnostics | 2496 | Secondary diabetes mellitus with neurological manifestations |
| cmbdh_diagnostics | 24970 | Secondary diabetes mellitus with peripheral circulatory disorders, not mentioned as uncontrolled or not specified |
| cmbdh_diagnostics | 25071 | Diabetes with peripheral circulatory disorders, type I [juvenile type], not listed as uncontrolled |
| cmbdh_diagnostics | 24901 | Secondary diabetes mellitus without mention of complication, uncontrolled |
| cmbdh_diagnostics | 25003 | Diabetes mellitus without mention of complication, type I [juvenile type], uncontrolled |
| cmbdh_diagnostics | 2492 | Secondary diabetes mellitus with hyperosmolarity |
| cmbdh_diagnostics | 25021 | Diabetes with hyperosmolarity, type I [juvenile type], not mentioned as uncontrolled |
| cmbdh_diagnostics | 24990 | Secondary diabetes mellitus with unspecified complication, not mentioned as uncontrolled or unspecified |
| cmbdh_diagnostics | 25063 | Diabetes with neurological manifestations, type I [juvenile type], uncontrolled |
| cmbdh_diagnostics | 25010 | Diabetes with ketoacidosis, type II or type not specified, not mentioned as uncontrolled |
| cmbdh_diagnostics | 25083 | Diabetes with other specified manifestations, type I [juvenile type], uncontrolled |
| cmbdh_diagnostics | 24931 | Diabetes mellitus secondary to other types of coma, uncontrolled |
| cmbdh_diagnostics | 24900 | Secondary diabetes mellitus without mention of complication, not mentioned as uncontrolled or not specified |
| cmbdh_diagnostics | 24940 | Secondary diabetes mellitus with renal manifestations, not mentioned as uncontrolled or not specified |
| cmbdh_diagnostics | 2497 | Secondary diabetes mellitus with peripheral circulatory disorders |
| cmbdh_diagnostics | 24971 | Secondary diabetes mellitus with peripheral circulatory disorders, uncontrolled |
| cmbdh_diagnostics | 25053 | Diabetes with ophthalmic manifestations, type I [juvenile type], uncontrolled |
| cmbdh_diagnostics | 2493 | Diabetes mellitus secondary to other types of coma |
| cmbdh_diagnostics | 25011 | Diabetes with ketoacidosis, type I [juvenile type], not listed as uncontrolled |
| cmbdh_diagnostics | 25091 | Diabetes with unspecified complication, type I [juvenile type], not listed as uncontrolled |
| cmbdh_diagnostics | 24991 | Secondary diabetes mellitus with unspecified, uncontrolled complication |
| cmbdh_diagnostics | 24921 | Secondary diabetes mellitus with hyperosmolarity, uncontrolled |
| cmbdh_diagnostics | 25033 | Diabetes with another type of coma, type I [juvenile type], uncontrolled |
| cmbdh_diagnostics | 2491 | Diabetes mellitus secondary to ketoacidosis |
| cmbdh_diagnostics | 2495 | Secondary diabetes mellitus with ophthalmic manifestations |
| cmbdh_diagnostics | 24951 | Secondary diabetes mellitus with ophthalmic manifestations, uncontrolled |
| cmbdh_diagnostics | 249 | Secondary diabetes mellitus |
| cmbdh_diagnostics | 25093 | Diabetes with unspecified complication, type I [juvenile type], uncontrolled |
| cmbdh_diagnostics | 2499 | Secondary diabetes mellitus with unspecified complication |
| cmbdh_diagnostics | 25051 | Diabetes with ophthalmic manifestations, type I [juvenile type], not mentioned as uncontrolled |
| cmbdh_diagnostics | 25031 | Diabetes with another type of coma, type I [juvenile type], not mentioned as uncontrolled |
| cmbdh_diagnostics | 24980 | Diabetes mellitus secondary to other specified manifestations, not mentioned as uncontrolled or unspecified |
| cmbdh_diagnostics | 24911 | Diabetes mellitus secondary to ketoacidosis, uncontrolled |
| cmbdh_diagnostics | 24960 | Secondary diabetes mellitus with neurological manifestations, not mentioned as uncontrolled or not specified |
| cmbdh_diagnostics | 25013 | Diabetes with ketoacidosis, type I [juvenile type], uncontrolled |
| cmbdh_diagnostics | 25073 | Diabetes with peripheral circulatory disorders, type I [juvenile type], uncontrolled |
| cmbdh_diagnostics | 2494 | Secondary diabetes mellitus with renal manifestations |
| cmbdh_diagnostics | 24950 | Secondary diabetes mellitus with ophthalmic manifestations, not mentioned as uncontrolled or not specified |
| cmbdh_diagnostics | 25043 | Diabetes with renal manifestations, type I [juvenile type], uncontrolled |
| cmbdh_diagnostics | 2498 | Diabetes mellitus secondary to other specified manifestations |
| cmbdh_diagnostics | 2501 | Diabetes with ketoacidosis |
| cmbdh_diagnostics | 25061 | Diabetes with neurological manifestations, type I [juvenile type], not mentioned as uncontrolled |
| cmbdh_diagnostics | 25081 | Diabetes with other specified manifestations, type I [juvenile type], not mentioned as uncontrolled |
| cmbdh_diagnostics | 24981 | Diabetes mellitus secondary with other specified manifestations, uncontrolled |
| cmbdh_diagnostics | 24910 | Diabetes mellitus secondary to ketoacidosis, not listed as uncontrolled or not specified |
| cmbdh_diagnostics | 24961 | Secondary diabetes mellitus with neurological manifestations, uncontrolled |
| cmbdh_diagnostics | 24920 | Secondary diabetes mellitus with hyperosmolarity, not mentioned as uncontrolled or not specified |
| cmbdh_diagnostics | 25001 | Diabetes mellitus without mention of complication, type I [juvenile type], not mentioned as uncontrolled |
| cmbdh_diagnostics | 25012 | Diabetes with ketoacidosis, type II or type not specified, uncontrolled |
| cmbdh_diagnostics | 2490 | Secondary diabetes mellitus without mention of complication |
| cmbdh_diagnostics | 25023 | Diabetes with hyperosmolarity, type I [juvenile type], uncontrolled |
| cmbdh_diagnostics | 25011 | Diabetes with ketoacidosis, type I [juvenile type], not listed as uncontrolled |
| cmbdh_diagnostics | 25013 | Diabetes with ketoacidosis, type I [juvenile type], uncontrolled |
| cmbdh_diagnostics | 2500 | Diabetes mellitus without mention of complication |
| cmbdh_diagnostics | 25090 | Diabetes with unspecified complication, type II or type not specified, not mentioned as uncontrolled |
| cmbdh_diagnostics | 25052 | Diabetes with ophthalmic manifestations, type II or type not specified, uncontrolled |
| cmbdh_diagnostics | 25010 | Diabetes with ketoacidosis, type II or type not specified, not mentioned as uncontrolled |
| cmbdh_diagnostics | 2502 | Diabetes with hyperosmolarity |
| cmbdh_diagnostics | 25032 | Diabetes with another type of coma, type II or type not specified, uncontrolled |
| cmbdh_diagnostics | 25072 | Diabetes with peripheral circulatory disorders, type II or type not specified, uncontrolled |
| cmbdh_diagnostics | 25040 | Diabetes with renal manifestations, type II or type not specified, not mentioned as uncontrolled |
| cmbdh_diagnostics | 250 | Diabetes mellitus |
| cmbdh_diagnostics | 25002 | Diabetes mellitus without mention of complication, type II or type not specified, uncontrolled |
| cmbdh_diagnostics | 25020 | Diabetes with hyperosmolarity, type II or type not specified, not mentioned as uncontrolled |
| cmbdh_diagnostics | 25062 | Diabetes with neurological manifestations, type II or type not specified, uncontrolled |
| cmbdh_diagnostics | 25082 | Diabetes with other specified manifestations, type II or type not specified, uncontrolled |
| cmbdh_diagnostics | 25022 | Diabetes with hyperosmolarity, type II or unspecified type, uncontrolled |
| cmbdh_diagnostics | 25070 | Diabetes with peripheral circulatory disorders, type II or type not specified, not mentioned as uncontrolled |
| cmbdh_diagnostics | 25042 | Diabetes with renal manifestations, type II or type not specified, uncontrolled |
| cmbdh_diagnostics | 25060 | Diabetes with neurological manifestations, type II or type not specified, not mentioned as uncontrolled |
| cmbdh_diagnostics | 25000 | Diabetes mellitus without mention of complication, type II or type not specified, not mentioned as uncontrolled |
| cmbdh_diagnostics | 25080 | Diabetes with other specified manifestations, type II or type not specified, not mentioned as uncontrolled |
| cmbdh_diagnostics | 25050 | Diabetes with ophthalmic manifestations, type II or type not specified, not mentioned as uncontrolled |
| cmbdh_diagnostics | 25030 | Diabetes with another type of coma, type II or type not specified, not mentioned as uncontrolled |
| cmbdh_diagnostics | 25092 | Diabetes with unspecified complication, type II or type unspecified, uncontrolled |
| cmbdh_diagnostics | 25012 | Diabetes with ketoacidosis, type II or type not specified, uncontrolled |
| cmbdh_diagnostics | 29420 | Unspecified dementia, no conduct disorder |
| cmbdh_diagnostics | 29013 | Presenile dementia with depressive characteristics |
| cmbdh_diagnostics | 3311 | Frontotemporal dementia |
| cmbdh_diagnostics | 2903 | Senile dementia with delirium |
| cmbdh_diagnostics | 29040 | Uncomplicated vascular dementia |
| cmbdh_diagnostics | 29282 | Drug-induced persistent dementia |
| cmbdh_diagnostics | 29010 | Presenile dementia without complications |
| cmbdh_diagnostics | 2904 | Vascular dementia |
| cmbdh_diagnostics | 2900 | Uncomplicated senile dementia |
| cmbdh_diagnostics | 29021 | Senile dementia with depressive features |
| cmbdh_diagnostics | 29043 | Vascular dementia with depressive features |
| cmbdh_diagnostics | 33119 | Other frontotemporal dementias |
| cmbdh_diagnostics | 29421 | Unspecified dementia, with conduct disorder |
| cmbdh_diagnostics | 2912 | Alcohol-induced persistent dementia |
| cmbdh_diagnostics | 290 | Dementias |
| cmbdh_diagnostics | 2942 | Unspecified dementia |
| cmbdh_diagnostics | 29411 | Dementia in conditions classified elsewhere, with conduct disorder |
| cmbdh_diagnostics | 3310 | Alzheimer's disease |
| cmbdh_diagnostics | 29041 | Vascular dementia with delirium |
| cmbdh_diagnostics | 29012 | Presenile dementia with delusional characteristics |
| cmbdh_diagnostics | 2902 | Senile dementia with delusional or depressive characteristics |
| cmbdh_diagnostics | 33182 | Dementia with Lewy bodies |
| cmbdh_diagnostics | 29410 | Dementia in conditions classified elsewhere, without conduct disorder |
| cmbdh_diagnostics | 29011 | Presenile dementia with delirium |
| cmbdh_diagnostics | 2901 | Presenile dementia |
| cmbdh_diagnostics | 29020 | Senile dementia with delusional characteristics |
| cmbdh_diagnostics | 2941 | Dementia in conditions classified elsewhere |
| cmbdh_diagnostics | 29042 | Vascular dementia with delusional features |
| cmbdh_diagnostics | 30184 | Passive-aggressive personality |
| cmbdh_diagnostics | 29634 | Major severe depressive disorder of recurrent episodes, specified as accompanied by psychotic behavior |
| cmbdh_diagnostics | 29656 | Bipolar I disorder with the most recent (or current) depressive episode, in complete remission |
| cmbdh_diagnostics | 29615 | Manic disorder of recurrent episodes, in partial or unspecified remission |
| cmbdh_diagnostics | 30112 | Chronic depressive personality disorder |
| cmbdh_diagnostics | 29630 | Major depressive disorder of recurrent episodes, without specifying its severity |
| cmbdh_diagnostics | 29681 | Atypical manic disorder |
| cmbdh_diagnostics | 2968 | Other bipolar disorders and unspecified bipolar disorders |
| cmbdh_diagnostics | 29652 | Bipolar I moderate disorder with the most recent (or current) depressive episode |
| cmbdh_diagnostics | 29382 | Psychotic disorder with hallucinations in conditions classified elsewhere |
| cmbdh_diagnostics | 3016 | Dependent personality disorder |
| cmbdh_diagnostics | 29601 | Bipolar and mild disorder of single manic episode |
| cmbdh_diagnostics | 293 | Transient mental disorders caused by conditions classified elsewhere |
| cmbdh_diagnostics | 29623 | Major single-episode major depressive disorder, with no mention of psychotic behavior |
| cmbdh_diagnostics | 3012 | Schizoid personality disorder |
| cmbdh_diagnostics | 29605 | Bipolar I disorder of single manic episode, in partial or unspecified remission |
| cmbdh_diagnostics | 29644 | Bipolar and severe disorder with the most recent (or current) manic-type episode, specified as accompanied by psychotic behavior |
| cmbdh_diagnostics | 301 | Personality disorders |
| cmbdh_diagnostics | 29666 | Bipolar I disorder with the most recent (or current) episode of mixed type, in complete remission |
| cmbdh_diagnostics | 29662 | Bipolar I moderate disorder with the most recent (or current) mixed-type episode |
| cmbdh_diagnostics | 30120 | Unspecified schizoid personality disorder |
| cmbdh_diagnostics | 29612 | Moderate manic disorder of recurrent episodes |
| cmbdh_diagnostics | 29616 | Manic disorder of recurrent episodes, in complete remission |
| cmbdh_diagnostics | 30151 | Chronic factitious disease with physical symptoms |
| cmbdh_diagnostics | 30181 | Narcissistic personality disorder |
| cmbdh_diagnostics | 29640 | Bipolar I disorder with the most recent (or current) manic-type episode, without specifying its severity |
| cmbdh_diagnostics | 2961 | Manic disorder of recurrent episodes |
| cmbdh_diagnostics | 2965 | Bipolar I disorder with the most recent (or current) depressive type episode |
| cmbdh_diagnostics | 3019 | Unspecified personality disorder |
| cmbdh_diagnostics | 29680 | Bipolar disorder not specified |
| cmbdh_diagnostics | 3015 | Histrionic personality disorder |
| cmbdh_diagnostics | 2969 | Other episodic mood disorders and unspecified episodic mood disorders |
| cmbdh_diagnostics | 29635 | Major depressive disorder of recurrent episodes, in partial or unspecified remission |
| cmbdh_diagnostics | 29383 | Mood disorders in conditions classified elsewhere |
| cmbdh_diagnostics | 3011 | Affective personality disorder |
| cmbdh_diagnostics | 29653 | Bipolar and severe disorder with the most recent (or current) episode of a depressive type, without mention of psychotic behavior |
| cmbdh_diagnostics | 30159 | Other histrionic personality disorders |
| cmbdh_diagnostics | 30111 | Chronic hypomanic personality disorder |
| cmbdh_diagnostics | 30189 | Other personality disorders |
| cmbdh_diagnostics | 29631 | Mild major depressive disorder of recurrent episodes |
| cmbdh_diagnostics | 29622 | Moderate major depressive disorder single episode |
| cmbdh_diagnostics | 2931 | Subacute delirium caused by conditions classified elsewhere |
| cmbdh_diagnostics | 29600 | Bipolar I disorder with a single manic episode, without specifying its severity |
| cmbdh_diagnostics | 296 | Episodic mood disorders |
| cmbdh_diagnostics | 29626 | Major single-episode depressive disorder, in complete remission |
| cmbdh_diagnostics | 29604 | Bipolar and severe disorder of unique manic episode, specified as accompanied by psychotic behavior |
| cmbdh_diagnostics | 30121 | Introverted personality |
| cmbdh_diagnostics | 2962 | Major single-episode depressive disorder |
| cmbdh_diagnostics | 29665 | Bipolar I disorder with the most recent (or current) episode of mixed type, in partial or unspecified remission |
| cmbdh_diagnostics | 29643 | Bipolar and severe disorder with the most recent (or current) manic-type episode, with no mention of psychotic behavior |
| cmbdh_diagnostics | 29613 | Severe manic disorder of recurrent episodes, with no mention of psychotic behavior |
| cmbdh_diagnostics | 29661 | Mild bipolar I disorder with the most recent (or current) mixed-type episode |
| cmbdh_diagnostics | 2967 | Bipolar I disorder with the most recent (or current) episode of unspecified type |
| cmbdh_diagnostics | 30183 | Borderline personality disorder |
| cmbdh_diagnostics | 29614 | Severe manic disorder of recurrent episodes, specified as accompanied by psychotic behavior |
| cmbdh_diagnostics | 29655 | Bipolar I disorder with the most recent (or current) depressive episode, in partial or unspecified remission |
| cmbdh_diagnostics | 29663 | Bipolar and severe disorder with the most recent (or current) episode of mixed type, without mention of psychotic behavior |
| cmbdh_diagnostics | 30113 | Cyclothymic disorder |
| cmbdh_diagnostics | 3013 | Explosive personality disorder |
| cmbdh_diagnostics | 2960 | Bipolar I disorder with a single manic episode |
| cmbdh_diagnostics | 29624 | Major single-episode major depressive disorder, specified as accompanied by psychotic behavior |
| cmbdh_diagnostics | 29682 | Atypical depressive disorder |
| cmbdh_diagnostics | 29381 | Psychotic disorder with delusions in conditions classified elsewhere |
| cmbdh_diagnostics | 29641 | Mild bipolar I disorder with the most recent (or current) manic-type episode |
| cmbdh_diagnostics | 3017 | Antisocial personality disorder |
| cmbdh_diagnostics | 29645 | Bipolar I disorder with the most recent (or current) manic episode, in partial or unspecified remission |
| cmbdh_diagnostics | 29606 | Bipolar I disorder with a single manic episode, in complete remission |
| cmbdh_diagnostics | 29689 | Other bipolar disorders |
| cmbdh_diagnostics | 29651 | Mild bipolar I disorder with the most recent (or current) depressive type episode |
| cmbdh_diagnostics | 29699 | Other specified episodic mood disorders |
| cmbdh_diagnostics | 29620 | Major single-episode depressive disorder, without specifying its severity |
| cmbdh_diagnostics | 29611 | Mild manic disorder of recurrent episodes |
| cmbdh_diagnostics | 29389 | Other specified transient mental disorders caused by conditions classified elsewhere |
| cmbdh_diagnostics | 30182 | Avoidance personality disorder |
| cmbdh_diagnostics | 29602 | Bipolar and moderate disorder with a single manic episode |
| cmbdh_diagnostics | 29633 | Severe major depressive disorder of recurrent episodes, with no mention of psychotic behavior |
| cmbdh_diagnostics | 30150 | Unspecified histrionic personality disorder |
| cmbdh_diagnostics | 3018 | Other personality disorders |
| cmbdh_diagnostics | 2938 | Other specified transient mental disorders caused by conditions classified elsewhere |
| cmbdh_diagnostics | 29636 | Major depressive disorder of recurrent episodes, in complete remission |
| cmbdh_diagnostics | 3014 | Obsessive-compulsive personality disorder |
| cmbdh_diagnostics | 29646 | Bipolar I disorder with the most recent (or current) manic-type episode, in complete remission |
| cmbdh_diagnostics | 29625 | Major single-episode depressive disorder, in partial or unspecified remission |
| cmbdh_diagnostics | 2930 | Delirium caused by conditions classified elsewhere |
| cmbdh_diagnostics | 29610 | Manic disorder of recurrent episodes, without specifying its severity |
| cmbdh_diagnostics | 29650 | Bipolar I disorder with the most recent (or current) depressive episode, without specifying its severity |
| cmbdh_diagnostics | 30110 | Affective personality disorder not specified |
| cmbdh_diagnostics | 29621 | Mild single-episode major depressive disorder |
| cmbdh_diagnostics | 30122 | Schizotypal personality disorder |
| cmbdh_diagnostics | 29632 | Moderate major depressive disorder of recurrent episodes |
| cmbdh_diagnostics | 29603 | Bipolar and severe disorder of unique manic episode, without mention of psychotic behavior |
| cmbdh_diagnostics | 29660 | Bipolar I disorder with the most recent (or current) mixed-type episode, without specifying its severity |
| cmbdh_diagnostics | 3010 | Paranoid personality disorder |
| cmbdh_diagnostics | 29642 | Bipolar I moderate disorder with the most recent (or current) manic-type episode |
| cmbdh_diagnostics | 29690 | Unspecified episodic mood disorder |
| cmbdh_diagnostics | 2939 | Transient mental disorder not specified in conditions classified elsewhere |
| cmbdh_diagnostics | 29664 | Bipolar and severe disorder with the most recent (or current) mixed-type episode, specified as accompanied by psychotic behavior |
| cmbdh_diagnostics | 2963 | Major depressive disorder of recurrent episodes |
| cmbdh_diagnostics | 2966 | Bipolar I disorder with the most recent (or current) mixed-type episode |
| cmbdh_diagnostics | 29384 | Anxiety disorders in conditions classified elsewhere |
| cmbdh_diagnostics | 29654 | Bipolar and severe disorder with the most recent (or current) episode of depressive type, specified as accompanied by psychotic behavior |
| cmbdh_diagnostics | 2964 | Bipolar I disorder with the most recent (or current) manic-type episode |
| cmbdh_diagnostics | 402 | Hypertensive heart disease |
| cmbdh_diagnostics | 401 | Essential hypertension |
| cmbdh_diagnostics | 405 | Secondary hypertension |
| cmbdh_diagnostics | 4031 | Benign chronic hypertensive kidney disease |
| cmbdh_diagnostics | 40200 | Malignant hypertensive heart disease without heart failure |
| cmbdh_diagnostics | 4039 | Unspecified chronic hypertensive kidney disease |
| cmbdh_diagnostics | 40291 | Unspecified hypertensive heart disease with heart failure |
| cmbdh_diagnostics | 4059 | Unspecified secondary hypertension |
| cmbdh_diagnostics | 40511 | Renal benign secondary hypertension |
| cmbdh_diagnostics | 40301 | Malignant chronic hypertensive kidney disease with chronic stage V kidney disease or terminal kidney disease |
| cmbdh_diagnostics | 4051 | Benign secondary hypertension |
| cmbdh_diagnostics | 40519 | Other types of benign secondary hypertension |
| cmbdh_diagnostics | 4021 | Benign hypertensive heart disease |
| cmbdh_diagnostics | 40509 | Other types of secondary malignant hypertension |
| cmbdh_diagnostics | 40201 | Malignant hypertensive heart disease with heart failure |
| cmbdh_diagnostics | 4029 | Unspecified hypertensive heart disease |
| cmbdh_diagnostics | 40501 | Renovascular malignant secondary hypertension |
| cmbdh_diagnostics | 40300 | Malignant chronic hypertensive kidney disease with chronic kidney disease from stage I to stage IV or stage NE |
| cmbdh_diagnostics | 4010 | Malignant essential hypertension |
| cmbdh_diagnostics | 40599 | Other types of secondary hypertension not specified |
| cmbdh_diagnostics | 4030 | Malignant chronic hypertensive kidney disease |
| cmbdh_diagnostics | 40391 | Unspecified chronic hypertensive kidney disease with chronic stage V kidney disease or terminal kidney disease |
| cmbdh_diagnostics | 40311 | Benign chronic hypertensive kidney disease with chronic stage V kidney disease or terminal kidney disease |
| cmbdh_diagnostics | 40210 | Benign hypertensive heart disease without heart failure |
| cmbdh_diagnostics | 40401 | Hypertensive heart disease and chronic malignant hypertensive kidney disease with heart failure and chronic kidney disease from stage I to stage IV or stage NE |
| cmbdh_diagnostics | 40591 | Unspecified renovascular secondary hypertension |
| cmbdh_diagnostics | 40310 | Benign chronic hypertensive kidney disease with stage I to stage IV or stage NE chronic kidney disease |
| cmbdh_diagnostics | 40211 | Benign hypertensive heart disease with heart failure |
| cmbdh_diagnostics | 403 | Chronic hypertensive kidney disease |
| cmbdh_diagnostics | 4011 | Benign essential hypertension |
| cmbdh_diagnostics | 4019 | Essential hypertension not specified |
| cmbdh_diagnostics | 40290 | Unspecified hypertensive heart disease without heart failure |
| cmbdh_diagnostics | 4050 | Malignant secondary hypertension |
| cmbdh_diagnostics | 4020 | Malignant hypertensive heart disease |
| cmbdh_diagnostics | 40390 | Unspecified chronic hypertensive kidney disease with stage I to stage IV or stage NE chronic kidney disease |
| cmbdh_diagnostics | 42840 | Combined heart failure, systolic and diastolic, unspecified |
| cmbdh_diagnostics | 42832 | Chronic diastolic heart failure |
| cmbdh_diagnostics | 428 | Heart failure |
| cmbdh_diagnostics | 42823 | Acute chronic systolic heart failure |
| cmbdh_diagnostics | 42831 | Acute diastolic heart failure |
| cmbdh_diagnostics | 42841 | Combined heart failure, systolic and diastolic, acute |
| cmbdh_diagnostics | 4280 | Unspecified congestive heart failure |
| cmbdh_diagnostics | 4282 | Systolic heart failure |
| cmbdh_diagnostics | 42821 | Acute systolic heart failure |
| cmbdh_diagnostics | 42822 | Chronic systolic heart failure |
| cmbdh_diagnostics | 42843 | Combined heart failure, systolic and diastolic, chronic exacerbated |
| cmbdh_diagnostics | 4284 | Combined heart failure, systolic and diastolic |
| cmbdh_diagnostics | 4283 | Diastolic heart failure |
| cmbdh_diagnostics | 42833 | Acute chronic diastolic heart failure |
| cmbdh_diagnostics | 42820 | Unspecified systolic heart failure |
| cmbdh_diagnostics | 42842 | Combined heart failure, systolic and diastolic, chronic |
| cmbdh_diagnostics | 4281 | Left heart failure |
| cmbdh_diagnostics | 42830 | Unspecified diastolic heart failure |
| cmbdh_diagnostics | 4289 | Unspecified heart failure |
| cmbdh_diagnostics | 5845 | Acute renal failure with tubular necrosis injury |
| cmbdh_diagnostics | 5852 | Chronic kidney disease, stage II (mild) |
| cmbdh_diagnostics | 5849 | Acute renal failure unspecified |
| cmbdh_diagnostics | 5856 | Terminal renal disease |
| cmbdh_diagnostics | 584 | Acute renal failure |
| cmbdh_diagnostics | 5847 | Acute renal failure with renal medullary [papillary] necrosis injury |
| cmbdh_diagnostics | 5846 | Acute renal failure with renal cortical necrosis injury |
| cmbdh_diagnostics | 5851 | Chronic kidney disease, stage I |
| cmbdh_diagnostics | 5855 | Chronic kidney disease, stage V |
| cmbdh_diagnostics | 5859 | Unspecified chronic kidney disease |
| cmbdh_diagnostics | 5848 | Acute renal failure with other types of specified renal pathology |
| cmbdh_diagnostics | 5853 | Chronic kidney disease, stage III (moderate) |
| cmbdh_diagnostics | 585 | Chronic kidney disease |
| cmbdh_diagnostics | 586 | Unspecified renal failure |
| cmbdh_diagnostics | 5854 | Chronic kidney disease, stage IV (severe) |
| cmbdh_diagnostics | 4910 | Simple chronic bronchitis |
| cmbdh_diagnostics | 49121 | Chronic obstructive bronchitis with exacerbation (acute) |
| cmbdh_diagnostics | 49122 | Chronic obstructive bronchitis with acute bronchitis |
| cmbdh_diagnostics | 4911 | Chronic mucopurulent bronchitis |
| cmbdh_diagnostics | 491 | Chronic bronchitis |
| cmbdh_diagnostics | 49120 | Chronic obstructive bronchitis without exacerbation |
| cmbdh_diagnostics | 4912 | Chronic obstructive bronchitis |
| cmbdh_diagnostics | 3558 | Lower limb mononeuritis unspecified |
| cmbdh_diagnostics | 3540 | Carpal tunnel syndrome |
| cmbdh_diagnostics | 3548 | Other upper limb mononeuritis |
| cmbdh_diagnostics | 3549 | Upper limb mononeuritis not specified |
| cmbdh_diagnostics | 355 | Mononeuritis of the lower extremity of unspecified location |
| cmbdh_diagnostics | 3545 | Multiple mononeuritis |
| cmbdh_diagnostics | 3559 | Mononeuritis of unspecified location |
| cmbdh_diagnostics | 29503 | Subchronic simple schizophrenia with acute exacerbation |
| cmbdh_diagnostics | 29583 | Other specified types of subchronic schizophrenia with acute exacerbation |
| cmbdh_diagnostics | 2958 | Other specified types of schizophrenia |
| cmbdh_diagnostics | 29553 | Subchronic latent schizophrenia with acute exacerbation |
| cmbdh_diagnostics | 29544 | Chronic schizophreniform disorder with acute exacerbation |
| cmbdh_diagnostics | 2950 | Simple schizophrenia |
| cmbdh_diagnostics | 2954 | Schizophreniform disorder |
| cmbdh_diagnostics | 29540 | Schizophreniform disorder of unspecified course |
| cmbdh_diagnostics | 29573 | Subchronic schizoaffective disorder with acute exacerbation |
| cmbdh_diagnostics | 29531 | Subchronic paranoid schizophrenia |
| cmbdh_diagnostics | 29590 | Unspecified course unspecified schizophrenia |
| cmbdh_diagnostics | 29513 | Subchronic disorganized schizophrenia with acute exacerbation |
| cmbdh_diagnostics | 29594 | Chronic unspecified schizophrenia with acute exacerbation |
| cmbdh_diagnostics | 29560 | Residual schizophrenia of unspecified course |
| cmbdh_diagnostics | 29522 | Chronic catatonic schizophrenia |
| cmbdh_diagnostics | 29535 | Paranoid schizophrenia in remission |
| cmbdh_diagnostics | 29564 | Chronic residual schizophrenia with acute exacerbation |
| cmbdh_diagnostics | 2983 | Acute paranoid reaction |
| cmbdh_diagnostics | 2953 | Paranoid schizophrenia |
| cmbdh_diagnostics | 29552 | Chronic latent schizophrenia |
| cmbdh_diagnostics | 29500 | Simple schizophrenia of unspecified course |
| cmbdh_diagnostics | 29580 | Other specified types of unspecified course schizophrenia |
| cmbdh_diagnostics | 2957 | Schizoaffective disorder |
| cmbdh_diagnostics | 29504 | Chronic simple schizophrenia with acute exacerbation |
| cmbdh_diagnostics | 29584 | Other specified types of chronic schizophrenia with acute exacerbation |
| cmbdh_diagnostics | 29545 | Schizophreniform disorder in remission |
| cmbdh_diagnostics | 29541 | Subchronic schizophreniform disorder |
| cmbdh_diagnostics | 29572 | Chronic schizoaffective disorder |
| cmbdh_diagnostics | 2988 | Other reactive psychoses and unspecified reactive psychoses |
| cmbdh_diagnostics | 2984 | Psychogenic paranoid psychosis |
| cmbdh_diagnostics | 29593 | Subchronic unspecified schizophrenia with acute exacerbation |
| cmbdh_diagnostics | 29561 | Subchronic residual schizophrenia |
| cmbdh_diagnostics | 29512 | Chronic disorganized schizophrenia |
| cmbdh_diagnostics | 2980 | Depressive type psychosis |
| cmbdh_diagnostics | 29530 | Paranoid schizophrenia of unspecified course |
| cmbdh_diagnostics | 29554 | Chronic latent schizophrenia with acute exacerbation |
| cmbdh_diagnostics | 29502 | Chronic simple schizophrenia |
| cmbdh_diagnostics | 2951 | Disorganized schizophrenia |
| cmbdh_diagnostics | 29532 | Chronic paranoid schizophrenia |
| cmbdh_diagnostics | 29582 | Other specified types of chronic schizophrenia |
| cmbdh_diagnostics | 29543 | Subchronic schizophreniform disorder with acute exacerbation |
| cmbdh_diagnostics | 29574 | Chronic schizoaffective disorder with acute exacerbation |
| cmbdh_diagnostics | 29525 | Catatonic schizophrenia in remission |
| cmbdh_diagnostics | 29595 | Schizophrenia not specified in remission |
| cmbdh_diagnostics | 29550 | Latent schizophrenia of unspecified course |
| cmbdh_diagnostics | 29510 | Disorganized schizophrenia of unspecified course |
| cmbdh_diagnostics | 2955 | Latent schizophrenia |
| cmbdh_diagnostics | 29591 | Unspecified subchronic schizophrenia |
| cmbdh_diagnostics | 29570 | Schizoaffective disorder of course not specified |
| cmbdh_diagnostics | 29520 | Catatonic schizophrenia of unspecified course |
| cmbdh_diagnostics | 298 | Other non-organic psychoses |
| cmbdh_diagnostics | 2959 | Unspecified schizophrenia |
| cmbdh_diagnostics | 29523 | Subchronic catatonic schizophrenia with acute exacerbation |
| cmbdh_diagnostics | 29565 | Residual schizophrenia in remission |
| cmbdh_diagnostics | 29533 | Subchronic paranoid schizophrenia with acute exacerbation |
| cmbdh_diagnostics | 29551 | Subchronic latent schizophrenia |
| cmbdh_diagnostics | 29575 | Schizoaffective disorder in remission |
| cmbdh_diagnostics | 29505 | Simple schizophrenia in remission |
| cmbdh_diagnostics | 29511 | Subchronic disorganized schizophrenia |
| cmbdh_diagnostics | 29585 | Other specified types of schizophrenia in remission |
| cmbdh_diagnostics | 2981 | Exciting type psychosis |
| cmbdh_diagnostics | 29501 | Subchronic simple schizophrenia |
| cmbdh_diagnostics | 29581 | Other specified types of subchronic schizophrenia |
| cmbdh_diagnostics | 295 | Schizophrenic disorders |
| cmbdh_diagnostics | 2956 | Residual schizophrenia |
| cmbdh_diagnostics | 2989 | Unspecified psychosis |
| cmbdh_diagnostics | 29562 | Chronic residual schizophrenia |
| cmbdh_diagnostics | 29524 | Chronic catatonic schizophrenia with acute exacerbation |
| cmbdh_diagnostics | 29592 | Chronic unspecified schizophrenia |
| cmbdh_diagnostics | 2952 | Catatonic schizophrenia |
| cmbdh_diagnostics | 29542 | Chronic schizophreniform disorder |
| cmbdh_diagnostics | 29571 | Subchronic schizoaffective disorder |
| cmbdh_diagnostics | 29515 | Disorganized schizophrenia in remission |
| cmbdh_diagnostics | 29534 | Chronic paranoid schizophrenia with acute exacerbation |
| cmbdh_diagnostics | 29555 | Latent schizophrenia in remission |
| cmbdh_diagnostics | 29521 | Subchronic catatonic schizophrenia |
| cmbdh_diagnostics | 29563 | Subchronic residual schizophrenia with acute exacerbation |
| cmbdh_diagnostics | 29514 | Chronic disorganized schizophrenia with acute exacerbation |
| cmbdh_diagnostics | 2982 | Reactive confusion |
| diagnostics | I73.8 | OTHER SPECIFIC PERIPHERAL VASCULAR DISEASES |
| diagnostics | I73.9 | PERIPHERAL VASCULAR DISEASE, NOT SPECIFIED |
| diagnostics | I25.9 | CHRONIC ISCHEMIC HEART DISEASE, NOT SPECIFIED |
| diagnostics | I20.1 | BREAST ANGINA WITH DOCUMENTED SPASM |
| diagnostics | I25.5 | ISCHEMIC MYOCARDIOPATHY |
| diagnostics | I25.4 | ANONURISM OF THE CORONARY ARTERY |
| diagnostics | I25.6 | SILENT MYOCARDIUM ISCHEMIA |
| diagnostics | I25.8 | OTHER FORMS OF CHRONIC ISCHEMIC HEART DISEASE |
| diagnostics | I25 | CHRONIC ISCHEMIC HEART DISEASE |
| diagnostics | I20.9 | ANGINA DE PIT, NOT SPECIFIED |
| diagnostics | I20.8 | OTHER SPECIFIC FORMS OF BREAST ANGINA |
| diagnostics | I20 | ANGINA DE PIT |
| diagnostics | I20.0 | UNSTABLE ANGINA |
| diagnostics | I25.0 | ATHEROSCLEROTIC CARDIOVASCULAR DISEASE, DESCRIBED IN THIS WAY |
| diagnostics | I25.1 | ATHEROSCLEROTIC HEART DISEASE |
| diagnostics | I25.2 | ANCIENT MYOCARDIAL INFARCTION |
| diagnostics | I25.3 | HEART ANEURISM |
| diagnostics | E10.4 | INSULIN-DEPENDENT DIABETES MELLITUS, WITH NEUROLOGICAL COMPLICATIONS |
| diagnostics | E10.7 | INSULIN-DEPENDENT DIABETES MELLITUS, WITH MULTIPLE COMPLICATIONS |
| diagnostics | E12.3 | DIABETES MELLITUS ASSOCIATED WITH MALNUTRITION, WITH OPHTHALMOLOGICAL COMPLICATIONS |
| diagnostics | E13.6 | SPECIFIED DIABETES MELLITUS, WITH OTHER SPECIFIC COMPLICATIONS |
| diagnostics | E13.2 | SPECIFIED DIABETES MELLITUS, WITH KIDNEY COMPLICATIONS |
| diagnostics | E12.7 | DIABETES MELLITUS ASSOCIATED WITH MALNUTRITION, WITH MULTIPLE COMPLICATIONS |
| diagnostics | E13.0 | DIABETES MELLITUS SPECIFIED, WITH COMA |
| diagnostics | O24.4 | DIABETES MELLITUS ORIGINATED DURING PREGNANCY |
| diagnostics | E12.9 | DIABETES MELLITUS ASSOCIATED WITH MALNUTRITION, WITHOUT MENTION OF COMPLICATIONS |
| diagnostics | E13.3 | SPECIFIED DIABETES MELLITUS, WITH OPHTHALMOLOGICAL COMPLICATIONS |
| diagnostics | E13.4 | SPECIFIED DIABETES MELLITUS, WITH NEUROLOGICAL COMPLICATIONS |
| diagnostics | E13.5 | SPECIFIED DIABETES MELLITUS, WITH PERIPHERAL CIRCULATORY COMPLICATIONS |
| diagnostics | E13.7 | SPECIFIED DIABETES MELLITUS, WITH MULTIPLE COMPLICATIONS |
| diagnostics | E13.8 | DIABETES MELLITUS SPECIFIED, WITH NON-SPECIFIC COMPLICATIONS |
| diagnostics | E13.9 | DIABETES MELLITUS SPECIFIED, WITHOUT MENTION OF COMPLICATIONS |
| diagnostics | E12.5 | DIABETES MELLITUS ASSOCIATED WITH MALNUTRITION, WITH PERIPHERAL CIRCULATORY COMPLICATIONS |
| diagnostics | E12.6 | DIABETES MELLITUS ASSOCIATED WITH NUTRITION, WITH OTHER SPECIFIC COMPLICATIONS |
| diagnostics | E12.8 | DIABETES MELLITUS ASSOCIATED WITH NUTRITION, WITH NON-SPECIFIC COMPLICATIONS |
| diagnostics | E13 | OTHER NON-SPECIFIC DIABETES MELLITUS |
| diagnostics | E13.1 | SPECIFIED DIABETES MELLITUS, WITH KETOACIDOSIS |
| diagnostics | E12.4 | DIABETES MELLITUS ASSOCIATED WITH MALNUTRITION, WITH NEUROLOGICAL COMPLICATIONS |
| diagnostics | E12 | DIABETES MELLITUS ASSOCIATED WITH NUTRITION |
| diagnostics | E12.0 | DIABETES MELLITUS ASSOCIATED WITH NUTRITION, WITH COMA |
| diagnostics | E12.1 | DIABETES MELLITUS ASSOCIATED WITH NUTRITION, WITH KETOACIDOSIS |
| diagnostics | E12.2 | DIABETES MELLITUS ASSOCIATED WITH MALNUTRITION, WITH KIDNEY COMPLICATIONS |
| diagnostics | E10.9 | INSULIN-DEPENDENT DIABETES MELLITUS, WITHOUT MENTION OF COMPLICATIONS |
| diagnostics | E10.8 | INSULIN-DEPENDENT DIABETES MELLITUS, WITH NON-SPECIFIC COMPLICATIONS |
| diagnostics | E10.0 | INSULIN-DEPENDENT MELLITUS DIABETES, WITH COMA |
| diagnostics | E10.1 | INSULIN-DEPENDENT DIABETES MELLITUS, WITH KETOACIDOSIS |
| diagnostics | E10.2 | INSULIN-DEPENDENT MELLITUS DIABETES, WITH KIDNEY COMPLICATIONS |
| diagnostics | E10.3 | INSULIN-DEPENDENT DIABETES MELLITUS, WITH OPHTHALMOLOGICAL COMPLICATIONS |
| diagnostics | E10.5 | INSULIN-DEPENDENT DIABETES MELLITUS, WITH PERIPHERAL CIRCULATORY COMPLICATIONS |
| diagnostics | E10.6 | INSULIN-DEPENDENT DIABETES MELLITUS, WITH OTHER SPECIFIC COMPLICATIONS |
| diagnostics | E10 | DIABETES MELLITUS INSULINODEPENDENT |
| diagnostics | E11.1 | NON-INSULIN-DEPENDENT DIABETES MELLITUS, WITH KETOACIDOSIS |
| diagnostics | E11.2 | NON-INSULIN-DEPENDENT MELLITUS DIABETES WITH KIDNEY COMPLICATIONS |
| diagnostics | E11.5 | NON-INSULIN-DEPENDENT DIABETES MELLITUS, PERIPHERAL CIRCULATORY COMPLICATIONS |
| diagnostics | E14.9 | DIABETES MELLITUS, NOT SPECIFIED, WITHOUT MENTION OF COMPLICATIONS |
| diagnostics | E14.4 | DIABETES MELLITUS, NOT SPECIFIED, WITH NEUROLOGICAL COMPLICATIONS |
| diagnostics | E11.9 | NON-INSULIN-DEPENDENT DIABETES MELLITUS, WITHOUT MENTION OF COMPLICATIONS |
| diagnostics | E14.7 | DIABETES MELLITUS, NOT SPECIFIED, WITH MULTIPLE COMPLICATIONS |
| diagnostics | E14.1 | DIABETES MELLITUS, NOT SPECIFIED, WITH KETOACIDOSIS |
| diagnostics | E14.2 | MELLITUS DIABETES, NOT SPECIFIED, WITH KIDNEY COMPLICATIONS |
| diagnostics | E14.3 | DIABETES MELLITUS, NOT SPECIFIED, WITH OPHTHALMOLOGICAL COMPLICATIONS |
| diagnostics | E14.5 | DIABETES MELLITUS, NOT SPECIFIED, WITH PERIPHERAL CIRCULATORY COMPLICATIONS |
| diagnostics | E14.6 | DIABETES MELLITUS, NOT SPECIFIED, WITH OTHER SPECIFIC COMPLICATIONS |
| diagnostics | E14.8 | DIABETES MELLITUS, NOT SPECIFIED, WITH NON-SPECIFIC COMPLICATIONS |
| diagnostics | E14.0 | DIABETES MELLITUS, NOT SPECIFIED, WITH COMA |
| diagnostics | E14 | DIABETES MELLITUS, NON-SPECIFIC |
| diagnostics | E11.7 | NON-INSULIN-DEPENDENT DIABETES MELLITUS, WITH MULTIPLE COMPLICATIONS |
| diagnostics | E11.8 | NON-INSULIN-DEPENDENT DIABETES MELLITUS, WITH NON-SPECIFIC COMPLICATIONS |
| diagnostics | E11.6 | NON-INSULIN-DEPENDENT DIABETES MELLITUS, WITH OTHER SPECIFIC COMPLICATIONS |
| diagnostics | E11 | NON-INSULIN-DEPENDENT DIABETES MELLITUS |
| diagnostics | E11.0 | NON-INSULIN-DEPENDENT DIABETES MELLITUS, WITH COMA |
| diagnostics | E11.1 | NON-INSULIN-DEPENDENT DIABETES MELLITUS, WITH KETOACIDOSIS |
| diagnostics | E11.3 | NON-INSULIN-DEPENDENT DIABETES MELLITUS, WITH OPHTHALMOLOGICAL COMPLICATIONS |
| diagnostics | E11.4 | NON-INSULIN-DEPENDENT DIABETES MELLITUS, WITH NEUROLOGICAL COMPLICATIONS |
| diagnostics | F02.8 | DEMENTIA IN OTHER SPECIFIC DISEASES, CLASSIFIED IN OTHER PLACES |
| diagnostics | F01.1 | VASCULAR DEMENTIA FOR MULTIPLE HEART DISEASES |
| diagnostics | F00.1 | DEMENTIA IN ALZHEIMER'S DISEASE, OF LATE BEGINNING (G30.1 +) |
| diagnostics | F02.3 | DEMENTIA IN PARKINSON'S DISEASE (G20 +) |
| diagnostics | F01 | VASCULAR DEMENTIA |
| diagnostics | F02.2 | DEMENTIA IN HUNTINGTON'S DISEASE (GL0 +) |
| diagnostics | F06.7 | LIGHT COGNITIVE DISORDER |
| diagnostics | G31.0 | CIRCUMSCRIBED CEREBRAL ATROPHY |
| diagnostics | G31.1 | SENILE BRAIN DEGENERATION NOT CLASSIFIED ELSEWHERE |
| diagnostics | G31.2 | DEGENERATION OF THE NERVOUS SYSTEM CAUSED BY ALCOHOL |
| diagnostics | G31.8 | OTHER DEGENERATIVE DISEASES SPECIFIC TO THE NERVOUS SYSTEM |
| diagnostics | G31.9 | DEGENERATION OF THE NERVOUS SYSTEM, NON-SPECIFIC |
| diagnostics | G31 | OTHER DEGENERATIVE DISEASES OF THE NERVOUS SYSTEM IN DISEASES NOT CLASSIFIED ELSEWHERE |
| diagnostics | G30 | ALZHEIMER'S DISEASE |
| diagnostics | G30.0 | EARLY STARTING ALZHEIMER'S DISEASE |
| diagnostics | G30.1 | LATE BEGINNING ALZHEIMER'S DISEASE |
| diagnostics | G30.8 | OTHER TYPES OF LZHEIMER'S DISEASE |
| diagnostics | G30.9 | ALZHEIMER'S DISEASE, NOT SPECIFIED |
| diagnostics | F03 | DEMENTIA, NON-SPECIFIC |
| diagnostics | F01.3 | MIXED, CORTICAL AND SUBCORTICAL VASCULAR DEMENTIA |
| diagnostics | F01.8 | OTHER VASCULAR DEMENTIAS |
| diagnostics | F01.9 | VASCULAR DEMENTIA, NOT SPECIFIED |
| diagnostics | F02 | DEMENTIA IN OTHER DISEASES, CLASSIFIED IN OTHER PLACES |
| diagnostics | F02.0 | DEMENTIA IN PICK'S DISEASE (G3L.0 +) |
| diagnostics | F02.1 | DEMENTIA IN CREUTZFELDT-JAKOB DISEASE (A81.0 +) |
| diagnostics | F02.4 | DEMENTIA IN HUMAN IMMUNODEFICIENCY (HIV) VIRUS DISEASE (B22.0 +) |
| diagnostics | F01.2 | SUBCORTICAL VASCULAR DEMENTIA |
| diagnostics | F00 | DEMENTIA IN ALZHEIMER'S DISEASE (G30 .- +) |
| diagnostics | F00.0 | DEMENTIA IN ALZHEIMER'S DISEASE, EARLY BEGINNING (G30.0 +) |
| diagnostics | F00.2 | DEMENTIA IN ALZHEIMER'S DISEASE, ATYPICAL OR MIXED TYPE (G30.8 +) |
| diagnostics | F00.9 | DEMENTIA IN ALZHEIMER'S DISEASE, NOT SPECIFIED (G30.9 +) |
| diagnostics | F01.0 | ACUTE START VASCULAR DEMENTIA |
| diagnostics | F31.1 | BIPOLAR AFFECTIVE DISORDER, MANIC EPISODE PRESENT WITHOUT PSYCHOTIC SYMPTOMS |
| diagnostics | F38.1 | OTHER HUMOR DISORDERS (AFFECTIVE), RECURRING |
| diagnostics | F33.2 | RECURRENT DEPRESSIVE DISORDER, SERIOUS DEPRESSIVE EPISODE PRESENT WITHOUT PSYCHOTIC SYMPTOMS |
| diagnostics | F31.5 | BIPOLAR AFFECTIVE DISORDER, SERIOUS DEPRESSIVE EPISODE WITH PSYCHOTIC SYMPTOM |
| diagnostics | F32 | DEPRESSIVE EPISODE |
| diagnostics | F30 | MANIC EPISODE |
| diagnostics | F33.9 | RECURRENT DEPRESSIVE DISORDER, NOT SPECIFIED |
| diagnostics | F31.7 | BIPOLAR AFFECTIVE DISORDER, CURRENTLY IN REMISSION |
| diagnostics | F33.1 | RECURRENT DEPRESSIVE DISORDER, MODERATE EPISODE PRESENT |
| diagnostics | F39 | HUMOR DISORDER (AFFECTIVE DISORDER), NON-SPECIFIC |
| diagnostics | F34 | PERSISTENT HUMOR DISORDERS (AFFECTIVE) |
| diagnostics | F34.0 | CYCLOTHYMIA |
| diagnostics | F34.1 | DISTIMIA |
| diagnostics | F34.8 | OTHER PERSISTENT HUMOR DISORDERS (AFFECTIVE) |
| diagnostics | F34.9 | PERSISTENT HUMOR DISORDER (AFFECTIVE), NON-SPECIFIC |
| diagnostics | F38 | OTHER HUMOR DISORDERS (AFFECTIVE) |
| diagnostics | F38.0 | OTHER HUMOR DISORDERS (AFFECTIVE), ISOLATED |
| diagnostics | F33.8 | OTHER RECURRING DEPRESSIVE DISORDERS |
| diagnostics | F38.8 | OTHER HUMOR DISORDERS (AFFECTIVE), SPECIFIC |
| diagnostics | F32.3 | SERIOUS DEPRESSIVE EPISODE WITH PSYCHOTIC SYMPTOMS |
| diagnostics | F32.8 | OTHER DEPRESSIVE EPISODES |
| diagnostics | F32.9 | DEPRESSIVE EPISODE, NOT SPECIFIED |
| diagnostics | F33 | RECURRENT DEPRESSIVE DISORDER |
| diagnostics | F33.0 | RECURRENT DEPRESSIVE DISORDER, LIGHT EPISODE PRESENT |
| diagnostics | F33.3 | RECURRENT DEPRESSIVE DISORDER, SERIOUS DEPRESSIVE EPISODE PRESENT, WITH PSYCHOTIC SYMPTOM |
| diagnostics | F33.4 | RECURRING DEPRESSIVE DISORDER CURRENTLY IN REMISSION |
| diagnostics | F32.2 | SERIOUS DEPRESSIVE EPISODE WITHOUT PSYCHOTIC SYMPTOMS |
| diagnostics | F31.3 | BIPOLAR AFFECTIVE DISORDER, DEPRESSIVE EPISODE PRESENT Mild OR MODERATE |
| diagnostics | F31.4 | BIPOLAR AFFECTIVE DISORDER, SERIOUS DEPRESSIVE EPISODE WITHOUT PSYCHOTIC SYMPTOMS |
| diagnostics | F31.6 | BIPOLAR AFFECTIVE DISORDER, MIXED EPISODE PRESENT |
| diagnostics | F31.8 | OTHER BIPOLAR AFFECTIVE DISORDERS |
| diagnostics | F31.9 | BIPOLAR AFFECTIVE DISORDER, NOT SPECIFIED |
| diagnostics | F31.2 | BIPOLAR AFFECTIVE DISORDER, MANIC EPISODE PRESENT WITH PSYCHOTIC SYMPTOMS |
| diagnostics | F32.0 | LIGHT DEPRESSIVE EPISODE |
| diagnostics | F32.1 | MODERATE DEPRESSIVE EPISODE |
| diagnostics | F30.0 | HIPOMANIA |
| diagnostics | F30.1 | MANIA WITHOUT PSYCHOTIC SYMPTOMS |
| diagnostics | F30.2 | MANIA WITH PSYCHOTIC SYMPTOMS |
| diagnostics | F30.8 | OTHER MANIC EPISODES |
| diagnostics | F30.9 | MANIC EPISODE, NOT SPECIFIED |
| diagnostics | F31 | BIPOLAR AFFECTIVE DISORDER |
| diagnostics | F31.0 | BIPOLAR AFFECTIVE DISORDER, HYPOMANIC EPISODE PRESENT |
| diagnostics | F53.9 | PUERPERAL MENTAL DISORDER, NON-SPECIFIC |
| diagnostics | I11.9 | HYPERTENSIVE HEART DISEASE WITHOUT HEART FAILURE (CONGESTIVE) |
| diagnostics | I15.1 | HYPERTENSION SECONDARY TO OTHER KIDNEY DISORDERS |
| diagnostics | I12.9 | HYPERTENSIVE KIDNEY DISEASE WITHOUT KIDNEY FAILURE |
| diagnostics | I15.9 | SECONDARY HYPERTENSION, NOT SPECIFIED |
| diagnostics | I13.2 | HYPERTENSIVE HEART DISEASE WITH HEART FAILURE (CONGESTIVE) AND KIDNEY FAILURE |
| diagnostics | I13.9 | HYPERTENSIVE CARDIORENAL DISEASE, NOT SPECIFIED |
| diagnostics | I15 | SECONDARY HYPERTENSION |
| diagnostics | I15.0 | RENOVASCULAR HYPERTENSION |
| diagnostics | I15.2 | HYPERTENSION SECONDARY TO ENDOCRINE DISORDERS |
| diagnostics | I15.8 | OTHER TYPES OF SECONDARY HYPERTENSION |
| diagnostics | I13.1 | HYPERTENSIVE CARDIORENAL DISEASE WITH KIDNEY FAILURE |
| diagnostics | I10 | ESSENTIAL HYPERTENSION (PRIMARY) |
| diagnostics | I11 | HYPERTENSIVE HEART DISEASE |
| diagnostics | I11.0 | HYPERTENSIVE HEART DISEASE WITH HEART FAILURE (CONGESTIVE) |
| diagnostics | I12 | HYPERTENSIVE KIDNEY DISEASE |
| diagnostics | I12.0 | HYPERTENSIVE KIDNEY DISEASE WITH KIDNEY FAILURE |
| diagnostics | I13 | HYPERTENSIVE CARDIORENAL DISEASE |
| diagnostics | I13.0 | HYPERTENSIVE HEART DISEASE WITH HEART FAILURE (CONGESTIVE) |
| diagnostics | I50.1 | LEFT VENTRICULAR FAILURE |
| diagnostics | I50.9 | HEART FAILURE, NOT SPECIFIED |
| diagnostics | I50 | HEART FAILURE |
| diagnostics | I50.0 | CONGESTIVE HEART FAILURE |
| diagnostics | N18.9 | CHRONIC KIDNEY FAILURE, NOT SPECIFIED |
| diagnostics | N17.2 | ACUTE KIDNEY FAILURE WITH MEDULLARY NECROSIS |
| diagnostics | N17.8 | OTHER ACUTE KIDNEY FAILURES |
| diagnostics | N17.9 | ACUTE KIDNEY FAILURE, NOT SPECIFIED |
| diagnostics | N18 | CHRONIC KIDNEY FAILURE |
| diagnostics | N18.0 | TERMINAL KIDNEY FAILURE |
| diagnostics | N18.8 | OTHER CHRONIC KIDNEY FAILURES |
| diagnostics | N19 | NON-SPECIFIC KIDNEY FAILURE |
| diagnostics | N17 | ACUTE KIDNEY FAILURE |
| diagnostics | N17.0 | ACUTE KIDNEY FAILURE WITH TUBULAR NECROSIS |
| diagnostics | N17.1 | ACUTE KIDNEY FAILURE WITH ACUTE CORTICAL NECROSIS |
| diagnostics | J44.8 | OTHER SPECIFIC CHRONIC OBSTRUCTIVE LUNG DISEASES |
| diagnostics | J44.1 | CHRONIC OBSTRUCTIVE LUNG DISEASE, WITH ACUTE EXACERBATION, NOT SPECIFIED |
| diagnostics | J44.9 | CHRONIC OBSTRUCTIVE LUNG DISEASE, NOT SPECIFIED |
| diagnostics | G63.2 | DIABETIC POLYNEUROPATHY (E10-E14 + WITH FOURTH COMMON CHARACTER .4) |
| diagnostics | G99.0 | AUTONOMOUS NEUROPATHY IN METABOLIC AND ENDOCRINE DISEASES |
| diagnostics | E11.4 | NON-INSULIN-DEPENDENT DIABETES MELLITUS, WITH NEUROLOGICAL COMPLICATIONS |
| diagnostics | G59.0 | DIABETIC MONONEUROPATHY (E10-E14 + WITH FOURTH COMMON CHARACTER .4) |
| diagnostics | F25 | SCHIZOAFECTIVE DISORDERS |
| diagnostics | F23.1 | ACUTE POLYMORPHIC PSYCHOTIC DISORDER WITH SYMPTOMS OF SCHIZOPHRENIA |
| diagnostics | F22.9 | PERSISTENT DELIVERY DISORDER, NOT SPECIFIED |
| diagnostics | F22.8 | OTHER PERSISTENT DELUSIVE DISORDERS |
| diagnostics | F25.1 | SCHIZOAFFECTIVE DEPRESSIVE DISORDER |
| diagnostics | F20.0 | PARANOID SCHIZOPHRENIA |
| diagnostics | F22.0 | DELUSIVE DISORDER |
| diagnostics | F28 | OTHER PSYCHOTIC DISORDERS OF NON-ORGANIC ORIGIN |
| diagnostics | F29 | PSYCHOSIS OF NON-ORGANIC ORIGIN, NON-SPECIFIC |
| diagnostics | F25.9 | SCHIZOAFECTIVE DISORDER, NOT SPECIFIED |
| diagnostics | F23.2 | ACUTE PSYCHOTIC DISORDER OF SCHIZOPHRENIC TYPE |
| diagnostics | F23.3 | OTHER TYPES OF ACUTE PSYCHOTIC DISORDER, WITH A PREDOMINANCE OF DELUSIVE IDEAS |
| diagnostics | F23.8 | OTHER ACUTE AND TRANSITIONAL PSYCHOTIC DISORDERS |
| diagnostics | F23.9 | ACUTE AND TRANSIENT PSYCHOTIC DISORDER, NOT SPECIFIED |
| diagnostics | F24 | INDUCED DELUSIVE DISORDER |
| diagnostics | F25.0 | MANIAC-TYPE SCHIZOAFFECTIVE DISORDER |
| diagnostics | F25.2 | MIXED TYPE SCHIZOAFFECTIVE DISORDER |
| diagnostics | F25.8 | OTHER SCHIZOAFECTIVE DISORDERS |
| diagnostics | F20.4 | POSTESCHIZOPHRENIC DEPRESSION |
| diagnostics | F20.5 | RESIDUAL SCHIZOPHRENIA |
| diagnostics | F20.6 | SIMPLE SCHIZOPHRENIA |
| diagnostics | F20.8 | OTHER SCHIZOPHRENES |
| diagnostics | F20.9 | SCHIZOPHRENIA, NOT SPECIFIED |
| diagnostics | F21 | SCHIZOTYPIC DISORDER |
| diagnostics | F22 | PERSISTENT DELUSIVE DISORDERS |
| diagnostics | F20.3 | INDIFFERENTIATED SCHIZOPHRENIA |
| diagnostics | F23 | ACUTE AND TRANSITIONAL PSYCHOTIC DISORDERS |
| diagnostics | F23.0 | ACUTE POLYMORPHIC PSYCHOTIC DISORDER, WITHOUT SYMPTOMS OF SCHIZOPHRENIA |
| diagnostics | F20 | SCHIZOPHRENIA |
| diagnostics | F20.1 | HEBEPHRENIC SCHIZOPHRENIA |
| diagnostics | F20.2 | CATATHONIC SCHIZOPHRENIA |
| diagnostics | E78 | DISORDERS OF LIPOPROTEIN METABOLISM AND OTHER LIPIDEMIA |
| diagnostics | E78.0 | PURE HYPERCHOLESTEROLEMIA |
| diagnostics | E78.1 | PURE HYPERGLYCYRIDEMIA |
| diagnostics | E78.2 | MIXED HYPERLIPIDEMIA |
| diagnostics | E78.3 | HYPERQUILOMICRONEMIA |
| diagnostics | E78.4 | OTHER HYPERLIPIDEMIA |
| diagnostics | E78.5 | UNSPECIFIED HYPERLIPIDEMIA |
| diagnostics | E78.6 | LIPOPROTEIN DEFICIENCY |
| diagnostics | E78.8 | OTHER DISORDERS OF LIPOPROTEIN METABOLISM |
| diagnostics | E78.9 | LIPOPROTEIN METABOLISM DISORDER, NOT SPECIFIED |
| diagnostics | E11.3 | NON-INSULIN-DEPENDENT DIABETES MELLITUS, WITH OPHTHALMOLOGICAL COMPLICATIONS |
| diagnostics | E12.3 | DIABETES MELLITUS ASSOCIATED WITH MALNUTRITION, WITH OPHTHALMOLOGICAL COMPLICATIONS |
| diagnostics | E13.3 | SPECIFIED DIABETES MELLITUS, WITH OPHTHALMOLOGICAL COMPLICATIONS |
| diagnostics | E14.3 | DIABETES MELLITUS, NOT SPECIFIED, WITH OPHTHALMOLOGICAL COMPLICATIONS |
| diagnostics | H36.0 | DIABETIC RETINOPATHY (E10-E14 + WITH FOURTH COMMON CHARACTER.3) |
| diagnostics | G45.0 | VERTEBROBASILAR ARTERIAL SYNDROME |
| diagnostics | G45.1 | CAROTID ARTERY SYNDROME (HEMISPHERE) |
| diagnostics | G45.2 | BILATERAL AND MULTIPLE PRECEREBRAL ARTERIAL SYNDROMES |
| diagnostics | G45.3 | FLYING AMAUROSIS |
| diagnostics | G45.4 | TRANSITIONAL GLOBAL AMNESIA |
| diagnostics | G45.8 | OTHER TRANSITIONAL CEREBRAL ISCHEMIA AND RELATED SYNDROMES |
| diagnostics | G45.9 | TRANSIENT CEREBRAL ISCHEMIA, WITHOUT ANY OTHER SPECIFICATION |
| diagnostics | I67.8 | OTHER SPECIFIC CEREBROVASCULAR DISEASES |
| diagnostics | I67.9 | CEREBROVASCULAR DISEASE, NON-SPECIFIC |
| diagnostics | I60 | SUBARACHNOIDAL BLEEDING |
| diagnostics | I60.0 | SUBARACHNOIDAL SIPHON BLEEDING AND CAROTID BIFURCATION |
| diagnostics | I60.1 | SUBARACHNOIDAL HEMORRHAGE OF THE MIDDLE CEREBRAL ARTERY |
| diagnostics | I60.2 | SUBARACHNOIDAL BLEEDING OF THE ANTERIOR COMMUNICATING ARTERY |
| diagnostics | I60.3 | SUBARACHNOIDAL HEMORRHAGE OF THE REAR COMMUNICATING ARTERY |
| diagnostics | I60.4 | SUBARACHNOIDAL HEMORRHAGE OF THE BASILARY ARTERY |
| diagnostics | I60.5 | SUBARACHNOIDAL HEMORRHAGE OF THE VERTEBRAL ARTERY |
| diagnostics | I60.6 | SUBARACHNOIDAL HEMORRHAGE OF OTHER INTRACRANIAL ARTERIES |
| diagnostics | I60.7 | SUBARACHNOIDAL HEMORRHAGE OF AN UNSPECIFIED INTRACRANIAL ARTERY |
| diagnostics | I60.8 | OTHER SUBARACHNOIDAL BLEEDING |
| diagnostics | I60.9 | SUBARACHNOIDAL BLEEDING, NOT SPECIFIED |
| diagnostics | I61 | INTRAENCEPHAL HEMORRHAGE |
| diagnostics | I61.0 | INTRACEREBRAL HEMORRHAGE OF THE SUBCORTICAL HEMISPHERE |
| diagnostics | I61.1 | INTRACEREBRAL HEMORRHAGE OF THE CORTICAL HEMISPHERE |
| diagnostics | I61.2 | INTRACEREBRAL HEMORRHAGE OF THE HEMISPHERE, NOT SPECIFIED |
| diagnostics | I61.3 | INTRAENCEPHAL HEMORRHAGE IN THE BRAIN STEM |
| diagnostics | I61.4 | INTRAENCEPHAL HEMORRHAGE IN THE CEREBEL |
| diagnostics | I61.5 | INTRAENCEPHALIC, INTRAVENTRICULAR HEMORRHAGE |
| diagnostics | I61.6 | INTRAENCEPHALIC HEMORRHAGE OF MULTIPLE LOCATIONS |
| diagnostics | I61.8 | OTHER INTRAENCEPHAL BLEEDING |
| diagnostics | I61.9 | INTRAENCEPHAL BLEEDING, NOT SPECIFIED |
| diagnostics | I62 | OTHER NON-TRAUMATIC INTRACRANIAL BLEEDING |
| diagnostics | I62.9 | INTRACRANIAL (NON-TRAUMATIC) HEMORRHAGE, NOT SPECIFIED |
| diagnostics | I63 | CEREBRAL HEART ATTACK |
| diagnostics | I63.0 | CEREBRAL INFARCTION SECONDARY TO PRECEREBRAL ARTERY THROMBOSIS |
| diagnostics | I63.1 | CEREBRAL INFARCTION SECONDARY TO PRECEREBRAL ARTERY EMBOLIA |
| diagnostics | I63.2 | CEREBRAL INFARCTION SECONDARY TO OCCLUSION OR UNSPECIFIED STENOSIS OF PRECEREBRAL ARTERIES |
| diagnostics | I63.3 | CEREBRAL INFARCTION SECONDARY TO CEREBRAL ARTERY THROMBOSIS |
| diagnostics | I63.4 | CEREBRAL INFARCTION SECONDARY TO EMBOLIA B2265 CEREBRAL |
| diagnostics | I63.5 | CEREBRAL INFARCTION SECONDARY TO UNSPECIFIED OCCLUSION OR STENOSIS OF CEREBRAL ARTERIES |
| diagnostics | I63.6 | CEREBRAL INFARCTION SECONDARY TO CEREBRAL VEIN THROMBOSIS, DO NOT RAIN |
| diagnostics | I63.8 | OTHER BRAIN INFARCTIONS |
| diagnostics | I63.9 | CEREBRAL INFARCTION, NOT SPECIFIED |
| diagnostics | I64 | ACUTE, NON-SPECIFIC BRAIN VASCULAR ACCIDENT (HEMORRHAGIC OR ISCHEMIC GOOD) |
| diagnostics | I69 | SEQUELS OF CEREBROVASCULAR DISEASE |
| diagnostics | I69.0 | SEQUARIES OF SUBARACHNOIDAL HEMORRHAGE |
| diagnostics | I69.1 | SEQUENCES OF INTRAENCEPHAL BLEEDING |
| diagnostics | I69.2 | SEQUENCES OF OTHER NON-TRAUMATIC INTRACRANIAL BLEEDING |
| diagnostics | I69.3 | SEQUELS OF CEREBRAL INFARCTION |
| diagnostics | I69.4 | SEQUES OF CEREBRAL VASCULAR ACCIDENT NOT SPECIFIED AS BLEEDING OR HEART HEART |
| diagnostics | I69.8 | SEQUES OF OTHER CEREBROVASCULAR DISEASES AND UNSPECIFIED CEREBROVASCULAR DISEASES |
| farmacs_facturats | A10BH01 | Sitagliptin |
| farmacs_facturats | A10BH02 | Vildagliptin |
| farmacs_facturats | A10BH03 | Saxagliptin |
| farmacs_facturats | A10BH04 | Allogliptin |
| farmacs_facturats | A10BH05 | Linagliptin |
| farmacs_facturats | A10BH07 | Evogliptin |
| farmacs_facturats | A10BX09 | Dapagliflozin |
| farmacs_facturats | A10BX11 | Canagliflozin |
| farmacs_facturats | A10BX12 | Empagliflozin |
| farmacs_facturats | A10BK01 | Dapagliflozin |
| farmacs_facturats | A10BK03 | Empagliflozin |
| farmacs_facturats | A10BK02 | Canagliflozin |
| farmacs_facturats | A10BK04 | ERTUGLIFLOZINE |
| farmacs_facturats | A10BD10 | Metformin and Saxagliptin |
| farmacs_facturats | A10BD11 | Metformin and linagliptin |
| farmacs_facturats | A10BD13 | Metformin and Allogliptin |
| farmacs_facturats | A10BD08 | Metformin and vildagliptin |
| farmacs_facturats | A10BD07 | Metformin and sitagliptin |
| farmacs_facturats | A10BD15 | Metformin and Dapagliflozin |
| farmacs_facturats | A10BD16 | Metformin and canagliflozin |
| farmacs_facturats | A10BD20 | Metformin and Empagliflozin |
| farmacs_facturats | A10BD23 | metformin and ertugliflozin |
| farmacs_facturats | A10BD02 | Metformin and sulfonamides |
| farmacs_facturats | A10BB91 | Glisentida |
| farmacs_facturats | A10BB01 | Glibenclamide |
| farmacs_facturats | A10BB02 | Chlorpropamide |
| farmacs_facturats | A10BB03 | Tolbutamide |
| farmacs_facturats | A10BB04 | Glibornurida |
| farmacs_facturats | A10BB05 | Tolazamide |
| farmacs_facturats | A10BB06 | Carbutamide |
| farmacs_facturats | A10BB07 | Glipizida |
| farmacs_facturats | A10BB08 | Gliquidone |
| farmacs_facturats | A10BB09 | Gliclazide |
| farmacs_facturats | A10BB10 | Metahexamide |
| farmacs_facturats | A10BB11 | Glisoxepida |
| farmacs_facturats | A10BB12 | Glimepiride |
| farmacs_facturats | A10BB31 | Acetohexamide |
| farmacs_prescrits | A10BH01 | Sitagliptin |
| farmacs_prescrits | A10BH02 | Vildagliptin |
| farmacs_prescrits | A10BH03 | Saxagliptin |
| farmacs_prescrits | A10BH04 | Allogliptin |
| farmacs_prescrits | A10BH05 | Linagliptin |
| farmacs_prescrits | A10BH07 | Evogliptin |
| farmacs_prescrits | A10BX09 | Dapagliflozin |
| farmacs_prescrits | A10BX11 | Canagliflozin |
| farmacs_prescrits | A10BX12 | Empagliflozin |
| farmacs_prescrits | A10BK01 | Dapagliflozin |
| farmacs_prescrits | A10BK03 | Empagliflozin |
| farmacs_prescrits | A10BK02 | Canagliflozin |
| farmacs_prescrits | A10BK04 | ERTUGLIFLOZINE |
| farmacs_prescrits | A10BD10 | Metformin and Saxagliptin |
| farmacs_prescrits | A10BD11 | Metformin and linagliptin |
| farmacs_prescrits | A10BD13 | Metformin and Allogliptin |
| farmacs_prescrits | A10BD08 | Metformin and vildagliptin |
| farmacs_prescrits | A10BD07 | Metformin and sitagliptin |
| farmacs_prescrits | A10BD15 | Metformin and Dapagliflozin |
| farmacs_prescrits | A10BD16 | Metformin and canagliflozin |
| farmacs_prescrits | A10BD20 | Metformin and Empagliflozin |
| farmacs_prescrits | A10BD23 | metformin and ertugliflozin |
| farmacs_prescrits | A10BD02 | Metformin and sulfonamides |
| farmacs_prescrits | A10BB91 | Glisentida |
| farmacs_prescrits | A10BB01 | Glibenclamide |
| farmacs_prescrits | A10BB02 | Chlorpropamide |
| farmacs_prescrits | A10BB03 | Tolbutamide |
| farmacs_prescrits | A10BB04 | Glibornurida |
| farmacs_prescrits | A10BB05 | Tolazamide |
| farmacs_prescrits | A10BB06 | Carbutamide |
| farmacs_prescrits | A10BB07 | Glipizida |
| farmacs_prescrits | A10BB08 | Gliquidone |
| farmacs_prescrits | A10BB09 | Gliclazide |
| farmacs_prescrits | A10BB10 | Metahexamide |
| farmacs_prescrits | A10BB11 | Glisoxepida |
| farmacs_prescrits | A10BB12 | Glimepiride |
| farmacs_prescrits | A10BB31 | Acetohexamide |
| variables_analitiques | CAC | Albumin / Creatinine [mass / subst. (urine)] - LdRdC: |
| variables_analitiques | CKDEPI | Glomerular Filtration (CKD-EPI Estimation) - LdRdC: Estimated Glomerular Filtration (CKD-EPI) |
| variables_analitiques | COLHDL | HDL cholesterol [c.subst. (serum)] - LdRdC: HDL Cholesterol Serum / HDL / HDLc / c-HDL |
| variables_analitiques | COLLDL | LDL cholesterol [c.subst. (serum)] - LdRdC: LDL Cholesterol Serum / LDL / LDLc / c-LDL |
| variables_analitiques | COLTOT | Cholesterol [c.subst. (serum)] - LdRdC: Serum Cholesterol |
| variables_analitiques | CREAT | Creatinine [c.subst. (serum)] - LdRdC: Creatinine Serum / Creatininium / Creatinine Ion / Creatinemia |
| variables_analitiques | HBA1C | Glycohemoglobin (A1c) [fr.subst.] - LdRdC: Glycosylated Hemoglobin / HbA1c |
| variables_analitiques | TG | Triglyceride [c. subst. (serum)] - LdRdC: Serum Triglycerides / Triacylglycerols |
| variables_cliniques | TT103 | BMI (Body Mass Index) [kgs / m2] |
| variables_cliniques | EK202 | Diastolic Blood Pressure [mm Hg] |
| variables_cliniques | EK201 | Systolic Blood Pressure [mm Hg] |
| variables_cliniques | TT102 | Weight [kgs] / Peso / Weight |
| variables_cliniques | TT101 | Height [cm] / Height / Height |
